# The switchable phosphorescence and delayed fluorescence of a new rhodamine-like dye through allenylidene formation in a cyclometallated platinum(ii) system†‡

**DOI:** 10.1039/d1sc02787e

**Published:** 2021-07-07

**Authors:** Shunan Zhao, Yifan Zhu, Ling Li, Véronique Guerchais, Julien Boixel, Keith Man-Chung Wong

**Affiliations:** School of Chemistry and Chemical Engineering, Harbin Institute of Technology Harbin 15001 China; Department of Chemistry, Southern University of Science and Technology 1088 Xueyuan Blvd. Shenzhen 518055 P. R. China keithwongmc@sustech.edu.cn; Univ Rennes, CNRS UMR6226 F-3500 Rennes France veronique.guerchais@univ-rennes1.fr julien.boixel@univ-rennes1.fr

## Abstract

A new rhodamine-like alkyne-substituted ligand (Rhodyne) was designed to coordinate a cyclometallated platinum(ii) system. The chemo-induced “ON–OFF” switching capabilities on the spirolactone ring of the Rhodyne ligand with an end-capping platinum(ii) metal centre can modulate the interesting acetylide–allenylidene resonance. The long-lived ^3^IL excited state of Rhodyne in its ON state as an optically active opened form was revealed *via* steady-state and time-resolved spectroscopy studies. Exceptional near-infrared (NIR) phosphorescence and delayed fluorescence based on a rhodamine-like structure were observed at room temperature for the first time. The position of the alkyne communication bridge attached to the platinum(ii) unit was found to vary the lead(ii)-ion binding mode and also the possible resonance structure for metal-mediated allenylidene formation. The formation of a proposed allenylidene resonance structure was suggested to rationalize these phenomena.

## Abstract 2

A new rhodamine-like ligand (Rhodyne) was designed to coordinate a cyclometallated platinum(ii) system. Allenylidene formation could trigger NIR phosphorescence at 740 nm originating from Rhodyne ^3^IL, as well as delayed fluorescence at 620 nm.

## Graphical abstract

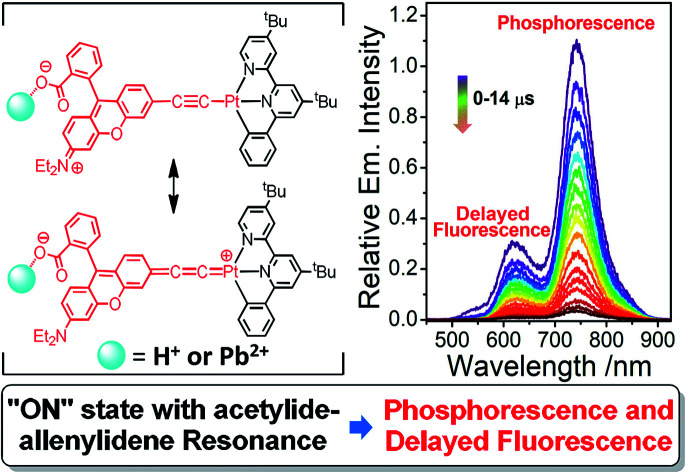

## Introduction

Transition metal complexes have attracted much interest due to a variety of potential applications,^1–7^ associated with the capability of promoting an emissive triplet excited state at room temperature.^8,9^ Particularly, the controllable population of the triplet excited state by an external stimulus has become an intense research topic of interest, dedicated to the examples of pH- and photo-activatable photodynamic therapy (PDT) reagents,^10–12^ molecular logic gates^13^ and data storage,^14^ or fundamental photochemistry.^15,16^ The incorporation of switchable organic units into optical-active transition metal complexes is one common strategy to achieve metal-based photo-functional materials with “ON” and “OFF” states in a controllable manner.^17–28^ The design of multi-chromophoric systems capable of triplet sensitization and emission with a switching unit could lead to remote manipulation of the localization and the lifetime of the triplet emissive state.

Rhodamine derivatives are found in equilibrium between two isomers with very different spectroscopic properties,^29^*i.e.* colourless and non-fluorescent ring-closed and highly coloured and fluorescent ring-opened structures about the spirocarbon. Because of this impressive attribute, together with their high sensitivity and selectivity, rhodamine derivatives have been widely applied in fluorescent probes.^29–32^ Although rhodamine was discovered over a century ago and widely investigated,^33^ most research about rhodamine has focused on its photophysics and photochemistry from the fluorescence of singlet excited states, studies about rhodamine triplet excited states are relatively limited.^34,35^ The introduction of a heavy atom into a rhodamine system acting as a photosensitizer has been employed to generate its triplet excited state for the application of photodynamic therapy.^36–39^ The research groups of Zhao^40,41^ and Eisenberg^42^ demonstrated that the attachment of a platinum(ii) system into the rhodamine unit would facilitate the formation of a rhodamine triplet excited state from its singlet excited state. Our group has recently developed a versatile strategy to generate a rhodamine triplet state as organelle-targeting photosensitizers for efficient PDT through the ligation of rhodamine tethered chelate into transition metal systems.^43,44^ Such a long-lived triplet excited state of rhodamine is commonly found in a dark state at room temperature which can only be observed by transient absorption spectroscopy.

**Chart 1:**
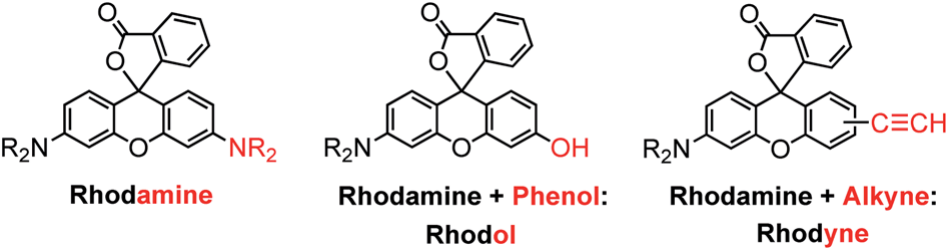
The molecular structures of rhodamine, Rhodol, and a new rhodamine-like dye with an alkyne substituent on the xanthene unit (named Rhodyne).

We have been devoted to the exploration of possible combinations of transition metal complexes with photo-switchable and chemo-switchable units, such as photochromic diarylethenes (DTE)^22–24^ and rhodamine-based derivatives,^43–48^ respectively. As an extension of our ongoing interest in controllable luminescence from transition metal systems, our previous results prompted us to design and investigate novel rhodamine-based cyclometallated platinum(ii) complexes. A structural modification for a rhodamine-like derivative is rationally designed as a new ligand, in which the triplet excited state of the rhodamine-like ligand could be promoted, while the switching behaviour could remain at the same time. In this study, an ethynyl group on the xanthene unit is introduced to replace the amino or hydroxyl group in rhodamine or Rhodol, respectively, for the coordination of the transition metal centre. Based on the naming of Rhodol with a hydroxyl group, we label this new rhodamine-like ligand as Rhodyne herein (Chart 1).

On the other hand, diverse examples of transition metal allenylidene complexes have been investigated because of their potential applications in catalysis^49^ and molecular electronics.^50^ On the contrary, the luminescence properties of this class of extended unsaturated complexes had been relatively unexplored until recent reports on the room-temperature phosphorescent allenylidene complexes of iridium(iii),^51^ gold(i),^52^ gold(iii)^53^ platinum(ii),^53^ and palladium(ii).^54^ In these systems, the alkylation or attachment of cationic N-heterocyclic carbene on the alkynyl group is a common strategy to achieve resonance forms of the allenylidene complex. The cationic Rhodyne in its opened form with the alkynyl linker at the 6′ position is anticipated to promote such resonance. As a result, the switching capability from the spirolactone ring on Rhodyne will be able to participate in the allenylidene formation in a controllable manner. Additionally, a higher metal contribution is envisaged to perturb the triplet excited state of the rhodamine-like dye through the delocalization of electron density over the main molecular skeleton, *i.e.* from the xanthene all the way to the metal centre across the allenylidene bridge, resulting from the extended resonance structure. In view of this, our design and approach in this regard is to tether the newly designed Rhodyne ligand to the cyclometallated platinum(ii) luminophore, with the aims of (1) introduction of a convenient and reversible switch into the platinum(ii) system to control the photophysical and photochemical properties; (2) exploration of the impact of the platinum(ii) centre on the photophysical properties of the rhodamine-like dye and (3) manipulation of the localization and the nature of an emissive triplet excited state in the hybrid from an organic dye and a luminescent transition metal system.

**Chart 2:**
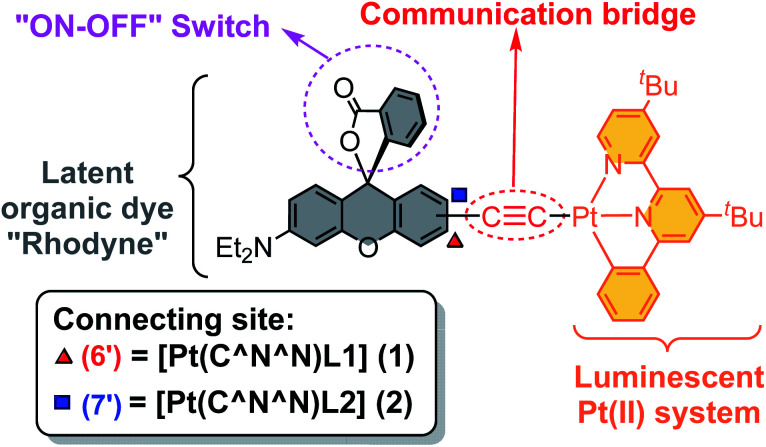
The structural design of the cyclometallated platinum(ii) complexes **1** and **2** with a switchable function rhodamine-like moiety.

As shown in Chart 2, two cyclometallated platinum(ii) complexes, comprising of Rhodyne as a rhodamine-like derivative switch, a luminescent platinum(ii) system and an alkynyl bridge, were designed. The ability to form an allenylidene complex, which may impact the luminescent behavior, would be inferred by the different positions of the alkynyl linker on Rhodyne. Herein we report the synthesis, characterization and spectroscopic studies of a new class of cyclometallated platinum(ii) complexes, **1** and **2**, with the Rhodyne ligand as the switchable regulator (Chart 2). The allenylidene complex formation in the opened-form of **1** with the alkynyl linker at a “suitable position” on Rhodyne is thought to be involved in the unusual rhodamine-like phosphorescence and delayed fluorescence.

## Results and discussion

### Synthesis and characterization

**Fig. 1:**
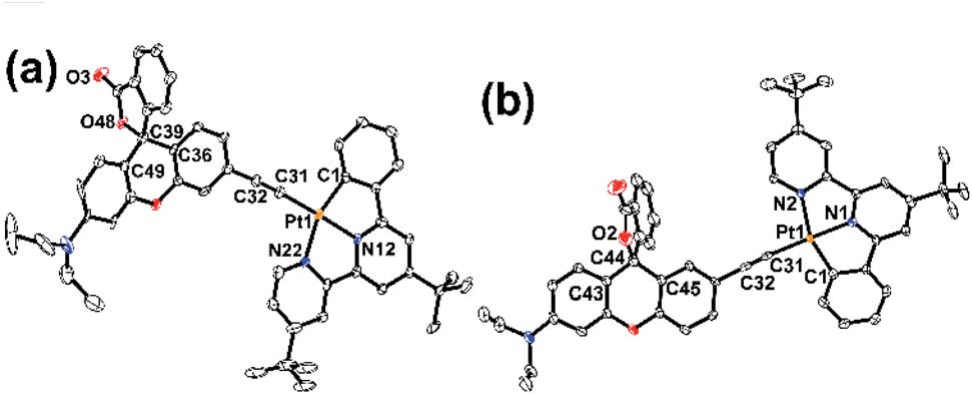
Perspective drawings of (a) **1** and (b) **2**. Solvent molecules and hydrogen atoms are omitted for clarity. Thermal ellipsoids are drawn at the 30% probability level.

In order to construct our designed cyclometallated platinum(ii) system, two new Rhodyne ligands with an alkynyl group at different positions, **L1** and **L2**, were firstly designed and synthesized (Scheme S1‡). The coordination of these two ligands into the cyclometallated chloroplatinum(ii) complex precursor was achieved in base conditions with the catalysis of CuI, to afford **1** and **2** (Scheme S1‡). Complexes **1** and **2** were fully characterized and their molecular structures determined by X-ray crystallography (Fig. S1–S4‡). As shown in their perspective views in Fig. 1, the platinum(ii) metal centre adopts a typical distorted square planar geometry with the cyclometallated N^N^C pincer ligand and the alkynyl linker. The geometry of the quaternary carbon of the spirolactone ring in both **1** and **2** clearly indicates the existence of the ring-closed form in the crystals.^29–32^ The angles between the xanthene core planes and the platinum(ii)-C^N^N framework are 12.12° and 34.31° in **1** and **2**, respectively.

### Basic photophysical properties in the closed form

Similar to related cyclometallated alkynylplatinum(ii) systems,^22,23,53,55^ low-energy absorption bands at 412 nm and 430 nm (**1**) and 420 nm and 442 nm (**2**) were observed in the electronic absorption spectra in acetonitrile at room temperature (Fig. S5a‡), which were assigned to the admixture of [dπ(Pt) → π*(N^N^C)] metal-to-ligand charge transfer (MLCT) and [pπ(C≡C) → π*(N^N^C)] ligand-to-ligand charge transfer (LLCT) transitions. Intense photoluminescence was also found in the acetonitrile solution of **1** (*λ*_max_ = 572 nm; *τ* = 215 and 660 ns) and **2** (*λ*_max_ = 598 nm; *τ* = 151 ns) (Fig. S5b‡), which originates from the triplet excited state of the ^3^MLCT/^3^LLCT character. The red-shift of the low-energy absorption and emission in **2**, relative to **1**, is ascribed to the stronger influence from the electron-rich alkoxy group at the *para*-position on the alkynyl ligand, leading to a rise of the HOMO energy level. A deoxygenated acetonitrile solution of **1** (3.1%) was found to show a higher photoluminescence quantum yield (PLQY) than **2** (1.9%). Their nanosecond transient absorption (ns-TA) different spectra (Fig. S6‡) show a positive absorption at around 372 nm which is assigned to the radical anion of N^N^C ligand absorption. In addition, the observation of a broad absorption from 450–800 nm extending beyond the detection range is attributed to the triplet absorption of the ^3^MLCT/^3^LLCT excited state.

### UV-vis absorption titration studies

**Fig. 2:**
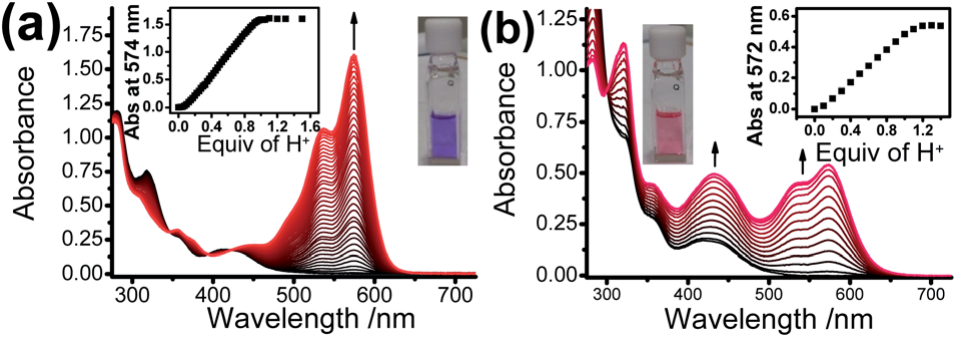
The electronic absorption spectral changes of (a) **1** (conc. = 10 μM) and (b) **2** (conc. = 25 μM) upon the addition of various equivalents of protons in acetonitrile at room temperature. The insets show plots of absorbance *versus* the concentration of protons and the solution colors in the opened forms.

Upon addition of *p*-toluene sulfonic acid, new structured absorption bands at 535 nm and 574 nm emerged in **1** as a result of the ring-opening process on Rhodyne, leading to a drastic colour change from yellow to purple in solution (Fig. 2a). In similar conditions, the ring-opened form of **2** obtained by addition of acid into the solution resulted in a colour change to magenta, which is attributed to the appearance of new structured absorption bands at 532 nm and 573 nm and an increase in absorbance at 432 nm (Fig. 2b). A similar amount of acid (1.1–1.2 equivalents of acid) was required to reach the absorbance plateau for the new absorption bands in **1** and **2** (Fig. 2 insets). The observation of a similar absorption response in **L1** and **L2** (Fig. S7‡) indicates the negligible influence from the alkynyl group at different substitution sites. In contrast, the absorption spectral changes upon ring opening in **1** show essential differences with those in **2**. Collectively, the differences in the acid response between them are as follows: (i) the change in the absorbance at around 420 nm in **1** was very tiny while the absorbance at 432 nm was found to increase from 0.16 to 0.50 in **2**; (ii) a much higher absorbance (around 6-fold) was generated in **1** for the newly emerged structured absorption band (from 500 to 600 nm) compared to that in **2**; (iii) two isosbestic points at 348 nm and 422 nm were observed in **1**, which were absent in **2**; and (iv) the shapes of the newly emerged absorption bands were different with Huang–Rhys factors of 0.66 (**1**) and 0.84 (**2**).

**Fig. 3:**
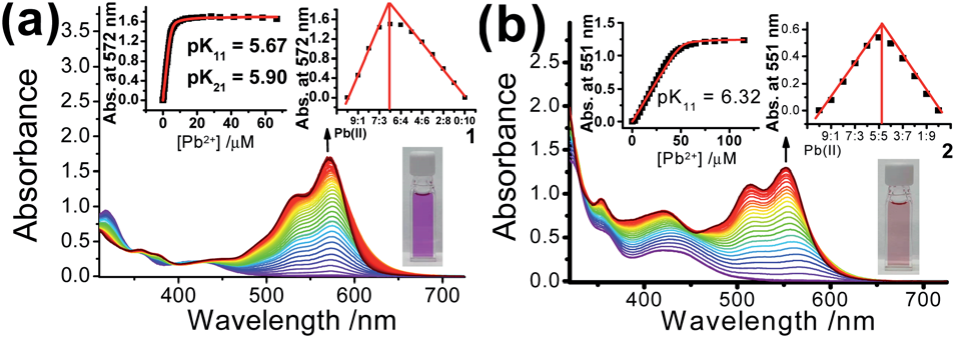
Electronic absorption spectral changes of (a) **1** (conc. = 10 μM) and (b) **2** (conc. = 25 μM) upon the addition of various concentrations of Pb(ii) ions in acetonitrile. The insets show plots of absorbance *versus* the concentration of Pb(ii) ions and the theoretical fitting curves; Job's plots; and the solution colours in the opened forms.

Apart from the acid-promoted ring-opening on the spirolactone ring of Rhodyne, a similar chemo-responsive process could also be triggered by metal ions in **1** and **2**. Corresponding absorption titration studies of **1** upon the addition of Pb(ii) ion exhibited similar spectral changes with new structured absorption bands, which are ascribed to the opened form of Rhodyne (Fig. 3a). Scrutinizing their spectral changes revealed that the absorption peak at 572 nm upon the addition of 0.5 equivalents of Pb(ii) ions was 83% as high as that at its saturation with about 1.2 equivalents of Pb(ii) ions (Fig. S8a‡). By monitoring the absorbance at the absorption tail at 620 nm, further addition of Pb(ii) ions beyond 0.67 equivalents of Pb(ii) ion leads to a decrease in the absorbance, suggesting a two-stage binding process (Fig. S8b‡). Both Job's plot and theoretical binding fitting (Fig. 3a insets) also supported the **1** : Pb(ii) ion = 2 : 1 binding model, where the initial addition of Pb(ii) ion afforded the {[**1**]_2_˙Pb^2+^} species which will be further converted into the {**1**˙Pb^2+^} adduct with excess Pb(ii) ions. The binding constants of 5.90 ± 0.13 (log *K*_21_) and 5.67 ± 0.21 (log *K*_11_) as well as the detection limit of 13.9 nM were determined. The normalized absorption spectrum of **1** with excess Pb(ii) ions matched very well with that in excess acid (Fig. S8b‡), indicating that the 1 : 1 bound species mainly exists in the presence of high concentrations of Pb(ii) ions. For **2**, new structured absorption bands at 520 nm and 567 nm emerged and the absorbance of the peak at *ca.* 430 nm increased upon the addition of Pb(ii) ions (Fig. 3b). In contrast, both the theoretical binding fitting and Job's plot (Fig. 3b insets) supported the 1 : 1 binding model. The detection limit of 76 nM and the log *K*_*s*_ value of 6.32 ± 0.17 for the Pb(ii) ion binding to **2** were found. The absence of 2 : 1 bound species in **2** is possibly due to the unfavourable formation of such a sandwich adduct as a result of stronger mutual repulsion from the bulky cyclometallated platinum(ii) moiety at the position in close proximity to the spirolactone group.

### Emission titration studies

Emission titration studies of **1** and **2** were carried out in air-saturated acetonitrile and the addition of acid or Pb(ii) ion resulted in similar spectral changes for the individual complex (Fig. S9 and S10‡). Upon excitation at the isosbestic point of 348 nm for **1** or at 330 nm for **2** (with minimal changes in absorbance), the original ^3^MLCT/LLCT emission in their closed forms was found to be diminished with the concentration of acid or Pb(ii) ions. Two residual emission band maxima (540 nm and 620 nm for **1**; 549 nm and 610 nm for **2**) were observed with an excess of acid (Fig. S9‡) or Pb(ii) ions (Fig. S10‡). The emission at 540 nm or 549 nm, for those excitation spectra are similar to the corresponding absorption spectra in the closed form, originating from the platinum(ii) ^3^MLCT/LLCT excited state. A related cyclometallated platinum(ii) complex with a positively charged alkynyl group was reported to exhibit similar ^3^MLCT/LLCT emission at 530 nm.^53^ The binding constants for **1** (log *K*_21_, 5.85; log *K*_11_, 6.11) and **2** (log *K*_11_, 6.62) are in good agreement with those determined by absorption studies, indicating that the emission quenching is attributed to the ring-opening process on Rhodyne.

**Fig. 4:**
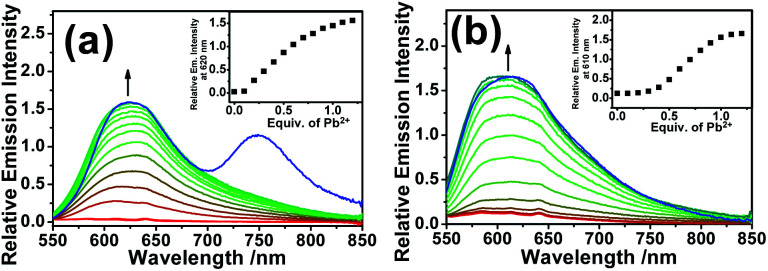
Emission spectral changes in acetonitrile upon the addition of various concentrations of Pb(ii) ions to (a) **1** and (b) **2** at an excitation wavelength of 540 nm; conc. = 10 μM. The insets show plots of the relative emission intensity *versus* the equivalents of Pb(ii) ions. Blue line: deoxygenated solution with an excess of Pb(ii) ions.

**Chart 3:**
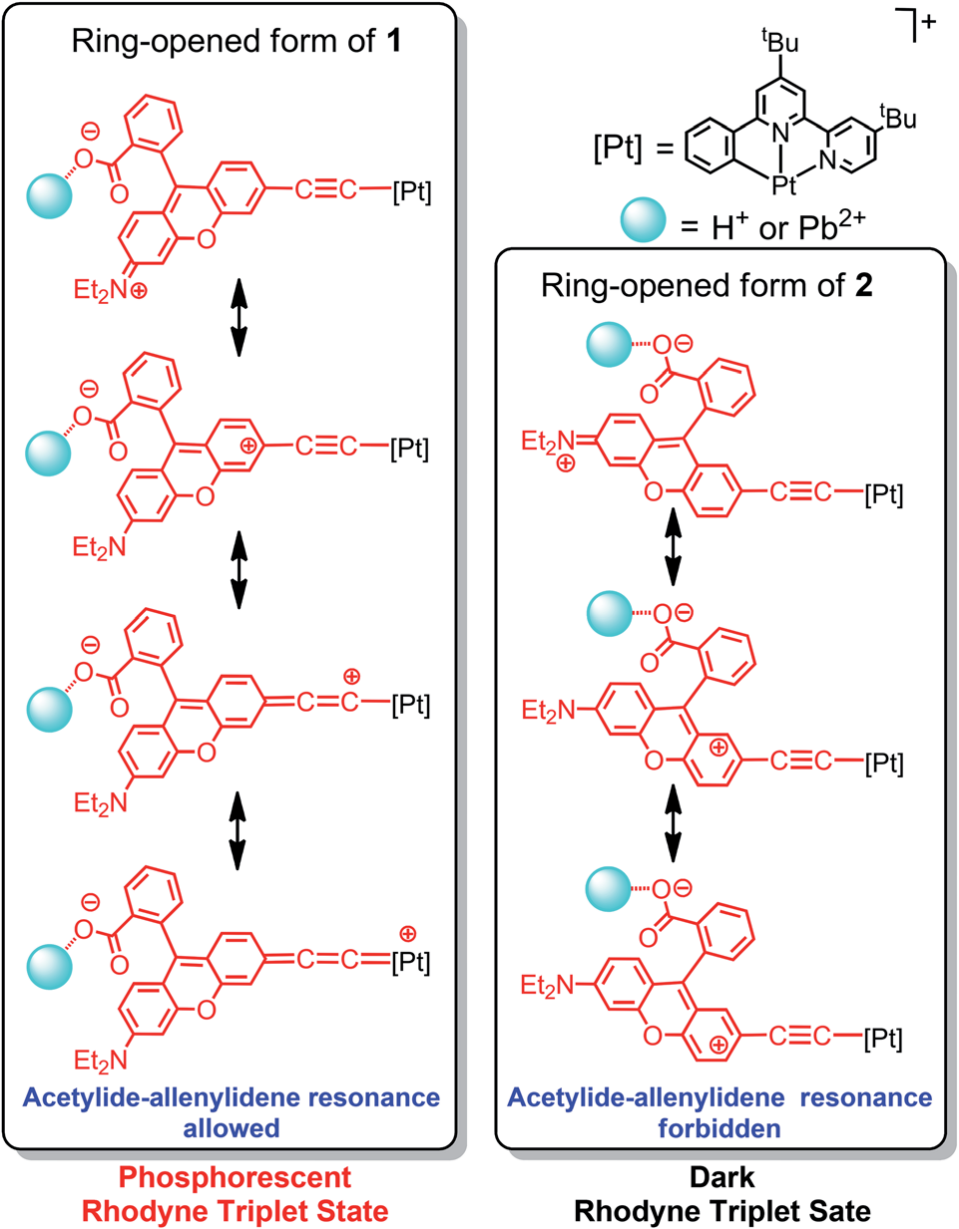
Resonance structures of **1** and **2** illustrating the possibility of platinum(ii) allenylidene formation through the positive charge created on Rhodyne in their ring-opened forms.

Since no platinum(ii) MLCT/LLCT state could be populated at such low-energy excitation, the ring-opening effect on the Rhodyne excited state is clearly identified. Upon excitation at 540 nm, new emission bands at 620 nm and 610 nm were found to gradually emerge with increasing the concentration of Pb(ii) ions in **1** and **2** (Fig. 4). The origin of these emissions is reasonably assigned as the Rhodyne singlet intraligand (^1^IL) excited state. It is noteworthy that a new near-infrared (NIR) emission band at *ca.* 750 nm appeared in the solution of **1** with excess Pb(ii) ions after removal of the oxygen (Fig. 4a). The excitation spectra of these emissions at 620 nm and 750 nm closely resemble the absorption spectrum of the ring-opened form of **1** featuring the characteristic Rhodyne ^1^IL absorption (Fig. S11‡), indicating that this emission originates from the opened form of Rhodyne. Given the large stokes shift of 4150 cm^−1^ (from 572 nm to 750 nm) as well as the nature of the oxygen sensitivity (Fig. 4a), the origin of such NIR emission is assigned as derived from the Rhodyne triplet intraligand (^3^IL) excited state. Kinetic studies of such NIR emission showing the lifetime in the microsecond regime (*vide infra*) further support the assignment of triplet parentage. Such phosphorescence of the ring-opened form of **1** was also observed in **1** with excess acid in deoxygenated acetonitrile. Similar to the triplet excited state of organic dyes, the rhodamine-based triplet excited state is commonly considered as a dark state and can be probed by transient absorption spectroscopy.^40–44^ To the best of our knowledge, this is the first example of direct observation of the rhodamine-like originated phosphorescence in solution state at room temperature. In contrast, such phosphorescence was not found in the deoxygenated solution of **2** in its ring-opened form. On the other hand, the emission responses of **L1** and **L2** upon ring opening are essentially the same (Fig. S12‡). On the basis of these findings, the position at which the alkynylplatinum(ii) unit is attached to the Rhodyne is suggested to be a key factor leading to a significant difference in the Rhodyne ^3^IL excited state properties in **1** and **2**. From consideration of the alkynylplatinum(ii) group which is attached to the 6′ position on Rhodyne in **1**, the positive charge created at a “suitable position” of the ring-opened form is envisaged to facilitate or contribute to the formation of the allenylidene complex with extended resonance throughout the xanthene core, allenylidene bridge and the platinum(ii) metal centre (Chart 3). On the contrary, the alkynylplatinum(ii) moiety in **2** is instead tethered at the 7′ position to the xanthene oxygen atom and the generation of the corresponding resonance structure is not allowed. It is reasonably suggested that the Rhodyne phosphorescence in the ring-opened form of **1** is mediated by such an allenylidene complex as a result of the enhanced involvement of the platinum(ii) metal centre.

### Computational studies

In order to elucidate the dependence of the alkynyl position tethered on the Rhodyne for the possibility of allenylidene formation, computational studies were performed. Optimized structures of **1** and **2** (Fig. S13a and b‡) revealed that the spiro-rings are in a closed state, as indicated by their X-ray crystal structures and photophysical behaviours. The opened forms of **1** and **2** were mimicked by replacing the isobenzofuran-1(3*H*)-one group with a phenyl ring and by adding one more positive charge throughout the whole structure (Fig. S13c and d‡). Compared to its closed form, it is noteworthy that both of the bond distances of Pt–C_α_ and C_β_–C_γ_ in the opened form of **1** are shortened by 0.02 Å, while the bond distance of C_α_–C_β_ is slightly elongated by 0.01 Å (Table S3‡). On the other hand, no such changes in the bond distances of Pt–C_α_ and C_β_–C_γ_ between the closed and opened forms of **2** were computed. Given the very similar structures of **1** and **2**, their discrepancies from the changes of bond distances are attributed to the variation of the connecting positions between Rhodyne and the alkynylplatinum(ii) moiety. For the opened form of **1**, the extent of the changes in the bond distances from the theoretical calculations is less significant than those in the iridium(iii) system possessing acetylide–allenylidene resonance structure.^51^ This can be rationalized by the less dominant allenylidene mesomeric structure in the opened form of **1** because the positive charge is delocalized mainly throughout the extended conjugation over the xanthene structure. The computational results reasonably support the formation of the allenylidene complex in the opened form of **1**. In sharp contrast, the corresponding allenylidene complex cannot be generated in the opened form of **2** due to the “unmatched” position of the alkynyl group on Rhodyne, as shown in Chart 3.

### Time-resolved emission and nanosecond transient absorption spectroscopy studies

**Fig. 5:**
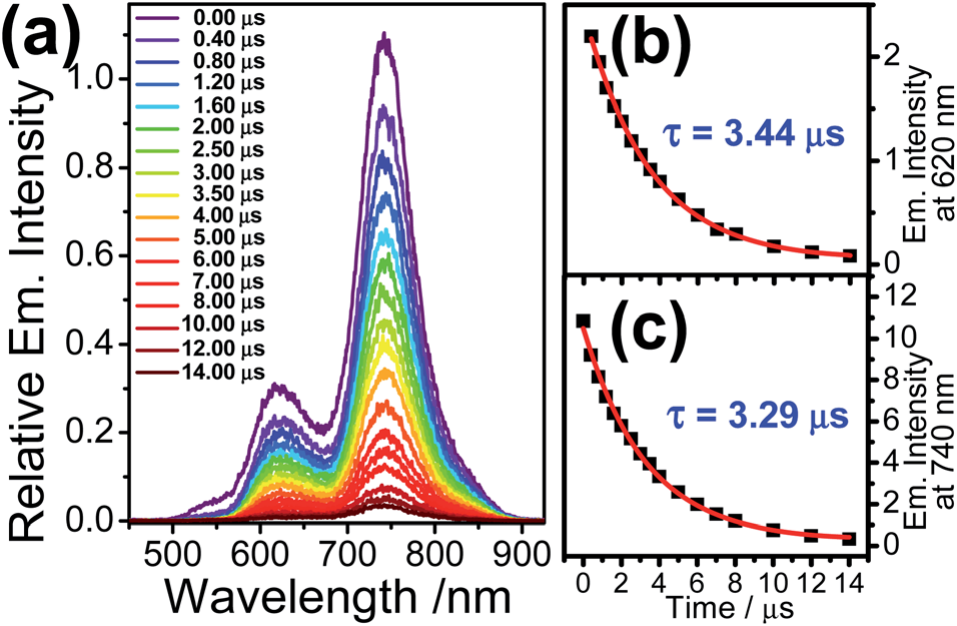
(a) Time-resolved emission spectra of **1** in the ring-opened form in deoxygenated acetonitrile at room temperature and the corresponding lifetime decay traces monitored at (b) 620 nm (with the exclusion of the first data point) and (c) 740 nm. Conc. = 15 μM (**1**) and 45 μM (Pb^2+^). A pulsed laser at 532 nm was the excitation source.

Nanosecond gated emission measurements of the ring-opened **1** at 532 nm-pulsed laser excitation were performed in order to obtain the emission dynamics from the Rhodyne excited sate, with a minimal influence from the ^3^MLCT state. The emission spectra in deoxygenated acetonitrile exhibit two emission bands at around 620 nm and 740 nm, while their relative emission intensities were found to be similar going from 0.0 to 14.0 μs after the laser pulse (Fig. 5a). Based on the aforementioned steady-state emission study of the ring-opened form of **1**, the emission bands at 620 nm and 740 nm originate from the Rhodyne singlet and triplet excited states, respectively. The emission lifetimes could be estimated by the decay fit of emission intensities at 620 nm and 740 nm (Fig. 5b and c). The long-lived NIR emission at 740 nm (*τ* = 3.29 μs) suggests the nature of the phosphorescence from the Rhodyne triplet excited state. This is further supported by the observation of long-lived species (*τ* = 3.39 μs) from the nanosecond transient absorption study (*vide infra*). It is noteworthy that the lifetime for the Rhodyne-based fluorescence at 620 nm was found to be unprecedentedly long (3.44 μs) and similar to the corresponding phosphorescence within experimental error. The prolonged lifetime suggests that such long-lived emission at 620 nm is attributed to the delayed fluorescence, while the absence of the short-lived Rhodyne prompted fluorescence is reasonably due to the cut-off from time zero setting from the instrument to eliminate the nanosecond laser pulse scattering. Such delayed fluorescence and phosphorescence of the opened form of **1** also emerged in deoxygenated dichloromethane (with 1% acetonitrile) (Fig. S14a‡) with longer lifetimes of *τ* = 12.88 μs and 13.19 μs (Fig. S14b and c‡), respectively. According to the proposed resonance structure in Chart 3, the d orbitals of the Pt centre can be involved in the IL transition through the linker. As a result, another MLCT (from Pt to Rhodyne) character is mixed with this IL state. This MLCT transition is probably less sensitive to the solvent polarity based on the similar time-resolved emission spectra in acetonitrile and dichloromethane (with 1% acetonitrile). On the other hand, computational studies showed that the contributions to the HOMO and LUMO in their lowest triplet state (T_1_) from the Pt center and C≡C group are higher in the opened form of **1** (Table S4 and Fig. S15‡), compared to that of **2**, which can further support certain electronic mixing between ^3^IL and ^3^MLCT states. In the air-saturated solution of the ring-opened **1** sample, the emission spectra from the pulsed laser excitation also showed the Rhodyne fluorescence and NIR phosphorescence at 620 nm and 740 nm, respectively (Fig. S16a‡). However, the emission lifetime at 740 nm was significantly shortened from 3.54 μs to 420 ns (Fig. S16b‡) in the air-saturated solution, which further confirms the triplet nature of the emission. The observation of the similar lifetime (390 ns) for the emission at 620 nm (Fig. S16c‡) also suggested the assignment of delayed fluorescence, which is essentially generated from the triplet state. For the open form of **2**, the main difference is the absence of phosphorescence from the rhodamine triplet excited state as well as the delayed fluorescence, probably due to the forbidden formation of the allenylidene complex.

Three possible mechanisms for delayed fluorescence, (I) triplet–triplet annihilation (TTA) with dominant phosphorescence or dominant delayed fluorescence, (II) recombination of geminately bound electron-hole pairs (GP) and (III) E-type delayed fluorescence or so-called thermally activated delayed fluorescence (TADF), have been reported.^56–58^ An attempt has been made to investigate the dependence of emission intensity on excitation laser fluence ranging from 1–213 mJ. The relative delayed fluorescence intensity to phosphorescence is similar with a ratio (*I*_DF_/*I*_Phos_) of about 0.27 (Fig. S17‡). However, no conclusive results could be obtained from the log–log plot of emission intensity *versus* laser power, probably due to the small changes in the emission intensity in the low laser power regime as well as the saturation effect in the high laser power regime. In spite of this, the aforementioned time-resolved measurements could still prove valuable and give some clues. By virtue of the lifetimes of the phosphorescence at 740 nm and the delayed fluorescence at 620 nm in the opened form of **1** being essentially the same, the assignment of delayed fluorescence from the TTA mechanism is unlikely because the lifetime of the delayed fluorescence from TTA should be half of the phosphorescence in this mechanism.^56^ In addition, a control experiment was conducted, in which time-resolved emission studies were carried out in viscous solvent, which was anticipated to avoid TTA delayed fluorescence because of the reduced molecular diffusion. The persistent observation of delayed fluorescence with a lifetime of 23.53 μs in glycerol triacetate (Fig. S18‡) was also inconsistent with the TTA mechanism. There was a report on the delayed fluorescence of rhodamine dyes coming from the GP recombination mechanism at 77 K, high energy excitation at 355 nm is required to populate the GP pair.^56^ In contrast, delayed fluorescence of the ring-closed form of **1** could also be observed from the lower energy of laser pulsed excitations at 532 nm. In addition, both the delayed fluorescence and phosphorescence were found to follow first-order kinetics with no power law relation, suggesting that the GP recombination mechanism is also less possible.^58^ Therefore, the origin of the delayed fluorescence is tentatively assigned to the TADF. In this mechanism, equal lifetimes of phosphorescence and delayed fluorescence would be suggested because the singlet and triplet state populations are in thermal equilibrium. Variable-temperature emission spectroscopic studies are a common way to verify the TADF mechanism. Upon increasing the temperature, enhanced delayed fluorescence intensity and decreased phosphorescence intensity would be anticipated due to the population of the singlet state from the triplet state through thermally activated process. However, the ring-opened form of **1** will undergo backwards ring-closure conversion in low temperature under −40 °C, the temperature ranging from −5 °C to 60 °C for variable-temperature emission measurement was therefore employed. The phosphorescence intensity at 740 nm was found to drop significantly upon increasing temperature, while the fluorescence intensity at 620 nm only slightly changed (Fig. S19a‡). From the basic knowledge of photophysics, the emission intensity would be weakened as a result of the increase in non-radiative decay with increasing temperature. From the normalized spectra at 740 nm, the relative fluorescence intensity to the phosphorescence is enhanced from −5 °C to 60 °C (Fig. S19b‡). Independent variable-temperature steady-state emission measurements for the opened form of **L1** showed that the fluorescence was significantly diminished upon increasing the temperature (Fig. S20‡). In view of this, another opposite effect from TADF is suggested to account for the small change in fluorescence intensity at 620 nm. The energy gap between the singlet and triplet states (Δ_S–T_) of about 0.39 eV was estimated from the onsets of fluorescence and phosphorescence energies, molecules in comparable Δ_S–T_ values were reported to exhibit TADF.^59,60^

**Fig. 6:**
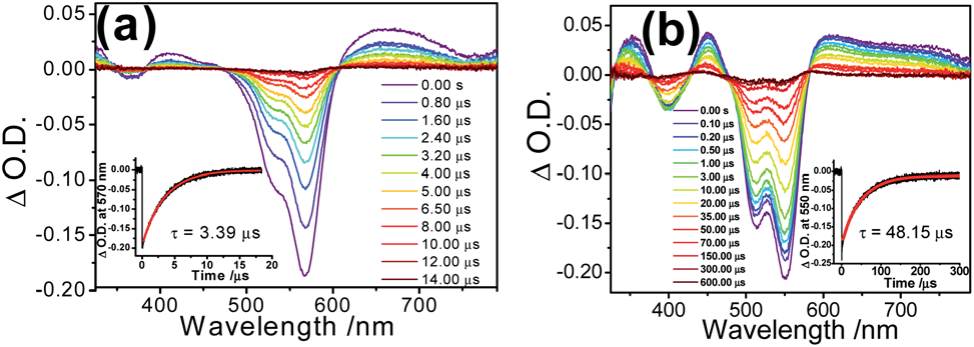
Transient absorption spectral changes of (a) **1** (conc. = 10 μM) and (b) **2** (conc. = 20 μM) in their ring-opened forms with excess amounts of Pb(ii) ions in deoxygenated acetonitrile at room temperature. The insets show the transient absorption lifetime fitting of the signals monitored at 570 nm and 550 nm for **1** and **2**, respectively. Excitation wavelength = 355 nm.

With reference to other related studies on rhodamine-containing transition metal systems,^41–44^ transient absorption (TA) spectroscopy is a valuable tool to probe the ^3^IL of rhodamine. In order to verify the excited state nature, nanosecond transient absorption (TA) spectroscopies of **1** and **2** in their ring-opened forms were investigated with the excitation of 355 nm pulsed laser. The TA spectra of the ring-opened form of **1** in deoxygenated acetonitrile displayed an intense ground state depletion signal at 570 nm, which is characteristic of the negative Rhodyne absorption profile (bleaching) (Fig. 6a). Such a ground state depletion signal at 570 nm with a lifetime of 3.39 μs is assigned as the Rhodyne ^3^IL state, which is in line with the lifetime of the phosphorescence at 740 nm. For the ring-opened form of **2**, a similar strong bleaching signal with a typical negative Rhodyne absorption profile was also found at around 550 nm in the TA spectrum (Fig. 6b), with a prolonged lifetime of 48.15 μs. Both in the opened forms of **1** and **2**, the observation of positive TA signals at 350 nm, 410–420 nm and 600–750 nm with similar lifetimes were ascribed to the ^3^IL absorption of Rhodyne.^41,42^ On the basis of the shorter lifetime of the Rhodyne ^3^IL state in **1**, a more efficient ^3^IL → S_0_ transition arising from the enhanced spin–orbit coupling is anticipated. This can be rationalized by the more substantial involvement or contribution of the platinum(ii) metal centre in the Rhodyne ^3^IL state due to the formation of the allenylidene complex in the opened form of **1**. Using a lower-energy wavelength of 532 nm pulsed laser for the excitation of the opened form of **1** also generated the same transient absorption signals in different solvents (Fig. S21‡). The lifetimes for the depletion signal at 570 nm (3.50 μs in MeCN; 12.83 μs in dichloromethane with 1% acetonitrile and 20.75 μs in glycerol triacetate) are in accordance with the phosphorescence lifetimes from the time-resolved emission spectroscopy.

The presence of the long-lived triplet excited state of Rhodyne is anticipated to interact with molecular oxygen (^3^O_2_) to form singlet oxygen (^1^O_2_). The ability of the opened forms of **1** and **2** to produce ^1^O_2_ was evaluated spectroscopically by the observation of ^1^O_2_ emission at about 1270 nm (Fig. S22‡). Moderate ^1^O_2_ generation quantum yields (*Φ*_Δ_) of 0.38 (**1**) and 0.35 (**2**) were determined by using rose Bengal as a reference (*Φ*_Δ_ in MeCN = 0.45)^61^ with excitation at 514.5 nm. Alternatively, the singlet oxygen generation abilities of both **1** and **2** in their ring-opened forms were studied using the ^1^O_2_ scavenger 1,3-diphenylisobenzofuran (DPBF) indicator. For both compounds, significant absorbance drops were observed at 410 nm (Fig. S23‡), indicating the generation of singlet oxygen.

**Fig. 7:**
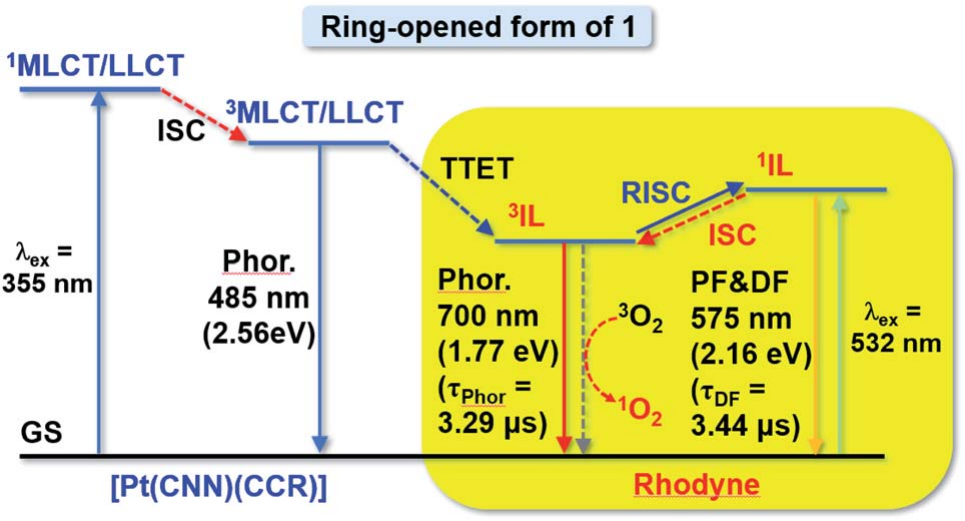
A simplified energy diagram of the ring-opened form of **1**. IL = intraligand excited state of Rhodyne; MLCT/LLCT = metal-to-ligand charge-transfer with mixing of ligand-to-ligand charge transfer character; a superscript of “1” = singlet state; a superscript of “3” = triplet state; ISC = intersystem crossing; RISC = reversed intersystem crossing; TTET = triplet–triplet energy transfer; PF = prompt fluorescence; DF: delayed fluorescence; and Phor. = phosphorescence. The energy levels were estimated based on the onsets of emission energies.

Based on the steady-state and time-resolved emission, as well as the nanosecond transient absorption spectroscopies in deoxygenated solvents, the simplified energy diagram of the ring-opened form of **1** with various excited states is suggested (Fig. 7). With excitation at around 348 nm (steady state measurements) or 355 nm (time-resolved studies) for the ^1^MLCT/LLCT transition of the platinum(ii) moiety of the ring-opened form of **1**, the emission intensity from the original ^3^MLCT/LLCT state diminished while Rhodyne ^1^IL fluorescence as well as the ^3^IL phosphorescence emerged. On the basis of this, together with our recent work on rhodamine-containing transition metal systems,^43,44^ the higher-lying platinum(ii) ^3^MLCT/LLCT state would be relaxed to the Rhodyne triplet intraligand (^3^IL) excited state *via* triplet–triplet energy transfer (TTET). The observation of residual ^3^MLCT/LLCT emission overlapping with the Rhodyne ^1^IL fluorescence with such a higher-energy excitation (Fig. S24‡) indicates that the TTET process is partially efficient. However, the possibility of direct population of the ^1^IL state from the ^3^MLCT state cannot be completely ruled out. If lower-energy excitation at 540 nm (steady state measurements) or 532 nm (time-resolved studies) was employed, the Rhodyne ^1^IL state was exclusively excited, which leads to the Rhodyne ^1^IL fluorescence and ^3^IL phosphorescence through intersystem crossing (ISC). It is noteworthy that delayed fluorescence, which is derived from the Rhodyne ^3^IL state through reversed intersystem crossing (RISC), was also observed from different excitation energies. Since the allenylidene complex formation is only possible in complex **1** with the alkynyl linker at a “suitable position” on Rhodyne, unusual rhodamine-like phosphorescence at 740 nm from its triplet state was observed at room temperature. In addition, an interesting delayed fluorescence at 620 nm of this rhodamine-like is also featured. Unlike the reports from Castellano and co-workers on transition metal systems with organic rylenes^62–64^ which exhibited an equilibrium between the ^3^MLCT and ^3^IL states, the delayed fluorescence in this work is derived from an equilibrium between the ^1^IL and ^3^IL states of Rhodyne. For the opened form of **2**, a higher-energy excitation for the ^1^MLCT/LLCT transition of a platinum(ii) moiety or lower-energy excitation for the Rhodyne ^1^IL state would also generate a Rhodyne triplet intraligand (^3^IL) excited state. Such a ^3^IL excited state is a dark state and could only be probed by nanosecond transient absorption spectroscopy. Unlike the ring-opened form of **1**, no phosphorescence was observed in that of **2** from the steady-state and time-resolved emission studies. In addition, the absence of delayed fluorescence from time-resolved emission measurements excludes the occurrence of RISC from the ^3^IL to ^1^IL state.

## Conclusions

In conclusion, two cyclometallated alkynylplatinum(ii) complexes tethered with rhodamine-like (Rhodyne) ligands showing controllable photophysical behaviour were described. *Via* the judicious choice of the tethering position on Rhodyne, the formation of an allenylidene complex can be generated upon the ring-opening of the spirolactone group. Specifically, NIR phosphorescence originating from Rhodyne ^3^IL at 740 nm and delayed fluorescence at 620 nm were featured in the solution state at room temperature. Such an observation of NIR phosphorescence and delayed fluorescence originating from a rhodamine-like structure at room temperature occurs for the first time, and it is mediated by allenylidene formation with a resonance structure throughout the xanthene core, an allenylidene bridge, and the platinum(ii) metal centre. The present study provides a strategic molecular design for lighting up the dark state of an organic excited state, not only providing new insight into fundamental photophysics but also allowing the exploitation of optical functional materials.

## Data availability

Data for this paper, including experimental and computational details, characterization, X-ray crystallographic information, other photophysical results are available at https://doi.org/10.1039/d0sc04751a.

## Author contributions

S. Z. contributed to synthesis and characterization, X-ray diffraction analysis, and optical measurements. Y. Z. contributed to the optical measurements. L. L. contributed to the DFT calculations. V. G. designed the project and performed data analysis. J. B. conducted the project, performed the synthesis and characterization, and prepared the manuscript. K. M. C. W. designed and conducted the project, performed data analysis, and prepared the manuscript.

## Conflicts of interest

There are no conflicts to declare.

## Supplementary Material

SC-012-D1SC02787E-s001

SC-012-D1SC02787E-s002

## References

[cit1] Zhao Q., Li F., Huang C. (2010). Chem. Soc. Rev..

[cit2] Baggaley E., Weinstein J. A., Williams J. A. G. (2012). Coord. Chem. Rev..

[cit3] Kamkaew A., Lim S. H., Lee H. B., Kiew L. V., Chung L. Y., Burgess K. (2013). Chem. Soc. Rev..

[cit4] Cakmak Y., Kolemen S., Duman S., Dede Y., Dolen Y., Kilic B., Kostereli Z., Yildirim L. T., Dogan A. L., Guc D., Akkaya E. U. (2011). Angew. Chem., Int. Ed..

[cit5] Wang C.-S., Dixneuf P. H., Soulé J.-F. (2018). Chem. Rev..

[cit6] Williams J. A. G., in Photochemistry and Photophysics of Coordination Compounds II, Springer Berlin Heidelberg, Berlin, Heidelberg, 2007, pp. 205–268

[cit7] Chi Y., Chou P.-T. (2010). Chem. Soc. Rev..

[cit8] Zhao J., Ji S., Wu W., Wu W., Guo H., Sun J., Sun H., Liu Y., Li Q., Huang L. (2012). RSC Adv..

[cit9] Zhao J., Wu W., Sun J., Guo S. (2013). Chem. Soc. Rev..

[cit10] Lovell J. F., Liu T. W. B., Chen J., Zheng G. (2010). Chem. Rev..

[cit11] McDonnell S. O., Hall M. J., Allen L. T., Byrne A., Gallagher W. M., O'Shea D. F. (2005). J. Am. Chem. Soc..

[cit12] Li B., Lin L., Lin H., Wilson B. C. (2016). J. Biophotonics.

[cit13] Erbas-Cakmak S., Akkaya E. U. (2013). Angew. Chem., Int. Ed..

[cit14] Mortimer R. J. and Rowley N. M., Metal Complexes as Dyes for Optical Data Storage and Electrochromic Materials, in Comprehensive Coordination Chemistry II, Pergamon, Oxford, 2003, pp. 581–619

[cit15] Zhao J., Xu K., Yang W., Wang Z., Zhong F. (2015). Chem. Soc. Rev..

[cit16] Cai S., Shi H., Li J., Gu L., Ni Y., Cheng Z., Wang S., Xiong W.-W., Li L., An Z., Huang W. (2017). Adv. Mater..

[cit17] Yam V. W. W., Lau V. C. Y., Cheung K. K. (1995). J. Chem. Soc., Chem. Commun..

[cit18] Yam V. W. W., Ko C. C., Wu L. X., Wong K. M. C., Cheung K. K. (2000). Organometallics.

[cit19] Ko C. C., Wu L. X., Wong K. M. C., Zhu N., Yam V. W. W. (2004). Chem.–Eur. J..

[cit20] Yam V. W. W., Ko C. C., Zhu N. (2004). J. Am. Chem. Soc..

[cit21] Ko C. C., Yam V. W. W. (2018). Acc. Chem. Res..

[cit22] Boixel J., Guerchais V., Le Bozec H., Jacquemin D., Amar A., Boucekkine A., Colombo A., Dragonetti C., Marinotto D., Roberto D., Righetto S., De Angelis R. (2014). J. Am. Chem. Soc..

[cit23] Boixel J., Zhu Y., Le Bozec H., Benmensour M. A., Boucekkine A., Wong K. M. C., Colombo A., Roberto D., Guerchais V., Jacquemin D. (2016). Chem. Commun..

[cit24] Zhao H., Garoni E., Roisnel R., Colombo A., Dragonetti C., Marinotto D., Righetto S., Roberto D., Jacquemin D., Boixel J., Guerchais V. (2018). Inorg. Chem..

[cit25] Tian H. and Zhang J., Photochromic Materials: Preparation, Properties and Applications, Wiley-VCH, Weinheim, Germany, 2016

[cit26] Tan W., Zhang Q., Zhang J., Tian H. (2009). Org. Lett..

[cit27] Belser P., de Cola L., Hartl F., Adamo V., Bozic B., Chriqui Y., Iyer V. M., Jukes R. T. F., Kühni J., Querol M., Roma S., Salluce N. (2006). Adv. Funct. Mater..

[cit28] Indelli M. T., Carli S., Ghirotti M., Chiorboli C., Ravaglia M., Garavelli M., Scandola F. (2008). J. Am. Chem. Soc..

[cit29] Beija M., Afonso C. A. M., Martinho J. M. G. (2009). Chem. Soc. Rev..

[cit30] Kim H. N., Lee M. H., Kim H. J., Kim J. S., Yoon J. (2008). Chem. Soc. Rev..

[cit31] Quang D. T., Kim J. S. (2010). Chem. Rev..

[cit32] Wang C., Wong K. M. C. (2013). Inorg. Chem..

[cit33] Noelting E., Dziewonski K. (1905). Ber. Dtsch. Chem. Ges..

[cit34] Cui X., Zhao J., Lou Z., Li S., Wu H., Han K. (2015). J. Org. Chem..

[cit35] Wang F., Cui X., Lou Z., Zhao J., Bao M., Li X. (2014). Chem. Commun..

[cit36] Calitree B., Donnelly D. J., Holt J. J., Gannon M. K., Nygren C. L., Sukumaran D. K., Autschbach J., Detty M. R. (2007). Organometallics.

[cit37] Hill J. E., Linder M. K., Davies K. S., Sawada G. A., Morgan J., Ohulchanskyy T. Y., Detty M. R. (2014). J. Med. Chem..

[cit38] Detty M. R., Prasad P. N., Donnelly D. J., Ohulchanskyy T., Gibson S. L., Hilf R. (2004). Bioorg. Med. Chem..

[cit39] Leonard K. A., Hall J. P., Nelen M. I., Davies S. R., Gollnick S. O., Camacho S., Oseroff A. R., Gibson S. L., Hilf R., Detty M. R. (2000). J. Med. Chem..

[cit40] Huang L., Zeng L., Guo H., Wu W., Wu W., Ji S., Zhao J. (2011). Eur. J. Inorg. Chem..

[cit41] Majumdar P., Cui X., Xu K., Zhao J. (2015). Dalton Trans..

[cit42] Li G., Mark M. F., Lv H., McCamant D. W., Eisenberg R. (2018). J. Am. Chem. Soc..

[cit43] Liu C. J., Zhou L. H., Wei F. F., Li L., Zhao S. N., Gong P., Cai L. T., Wong K. M. C. (2019). ACS Appl. Mater. Interfaces.

[cit44] Zhou L., Wei F., Xiang J., Li H., Li C., Zhang P., Liu C., Gong P., Cai L., Wong K. M. C. (2020). Chem. Sci..

[cit45] Wang C. Y., Wong K. M. C. (2011). Inorg. Chem..

[cit46] Wong K. M. C., Wang C. Y., Lam H. C., Zhu N. Y. (2015). Polyhedron.

[cit47] Wang C. Y., Lam H. C., Zhu N. Y., Wong K. M. C. (2015). Dalton Trans..

[cit48] Cheng Y. K., Li L., Wei F. F., Wong K. M. C. (2018). Inorg. Chem..

[cit49] Cadierno V., Gimeno J. (2009). Chem. Rev..

[cit50] Winter R. F., Záliš S. (2004). Coord. Chem. Rev..

[cit51] Kessler F., Curchod B. F. E., Tavernelli I., Rothlisberger U., Scopelliti R., Di Censo D., Gratzel M., Nazeeruddin Md. K., Baranoff E. (2012). Angew. Chem., Int. Ed..

[cit52] Xiao X.-S., Zou C., Guan X., Yang C., Lu W., Che C.-M. (2016). Chem. Commun..

[cit53] Xiao X.-S., Kwong W.-L., Guan X., Yang C., Lu W., Che C.-M. (2013). Chem.–Eur. J..

[cit54] Zou C., Lin J., Suo S., Xie M., Chang X., Lu W. (2018). Chem. Commun..

[cit55] Lu W., Mi B.-X., Chan M. C. W., Hui Z., Che C.-M., Zhu N., Lee S.-T. (2004). J. Am. Chem. Soc..

[cit56] Aydemir M., Jankus V., Dias F. B., Monkman A. (2014). Chem. Chem. Phys..

[cit57] Hayer A., Bässler H., Falk B., Schrader S. (2002). J. Phys. Chem. A.

[cit58] Chan K. T., Tong G. S. M., To W. P., Yang C., Du L., Phillips D. L., Che C.-M. (2017). Chem. Sci..

[cit59] Zhang Q., Li J., Shizu K., Huang S., Hirata S., Miyazaki H., Adachi C. (2012). J. Am. Chem. Soc..

[cit60] Dias F. B., Bourdakos K. N., Jankus V., Moss K. C., Kamtekar K. T., Bhalla V., Santos J., Bryce M. R., Monkman A. P. (2013). Adv. Mater..

[cit61] Stracke F., Heupel M., Thiel E. (1999). J. Photochem. Photobiol., A.

[cit62] Yarnell J. E., Deaton J. C., McCusker C. E., Castellano F. N. (2011). Inorg. Chem..

[cit63] Castellano F. N. (2012). Dalton Trans..

[cit64] Yarnell J. E., Wells K. A., Palmer J. R., Breaux J. M., Castellano F. N. (2019). J. Phys. Chem. B.

