# Supplementary material for: The switchable phosphorescence and delayed fluorescence of a new rhodamine-like dye through allenylidene formation in a cyclometallated platinum(ii) system
Source: Chem Sci. 2021 Jul 7;12(33):11056–64. doi: 10.1039/d1sc02787e (PMC8386667; doi:10.1039/d1sc02787e)
Supplement: SC-012-D1SC02787E-s001 [file SC-012-D1SC02787E-s001.pdf]

Electronic Supplementary Material (ESI) for Chemical Science.
This journal is © The Royal Society of Chemistry 2021

Switchable Phosphorescence and Delayed Fluorescence of a New Rhodamine-Like Dye Through the Allenylidene Formation in a Cyclometalated Platinum(II) System

Shunan Zhao,[a,b] Yifan Zhu,[b] Ling Li,[b] Véronique Guerchais,*[c] Julien Boixel,*[c] and Keith Man-Chung Wong*[b]

a School of Chemistry and Chemical Engineering, Harbin Institute of Technology, Harbin 15001, China

b Department of Chemistry, Southern University of Science and Technology, No. 1088, Tangchang Boulevard, Nanshan District, Shenzhen, Guangdong, P.R. China

Email: keithwongmc@sustech.edu.cn

c Dr. J. Boixel, Dr. V. Guerchais

Univ Rennes, CNRS UMR6226, F-3500 Rennes, France

E-mail: julien.boixel@univ-rennes1.fr; veronique.guerchais@univ-rennes1.fr

Supplementary Information

Materials and reagents. All the solvents for synthesis were of analytical grade. Acetonitrile for analysis was of spectrophotometric grade. 3-(Diethylamino)phenol, phthalic anhydride, 4-bromophenol, 3-bromophenol, potassium tetrachloroplatinate(II), 4,4'-di-*tert*-butyl-6-phenyl-2,2'-bipyridine, trimethylsilylacetylene, cuprous iodide, tetrabutylammonium fluoride were purchased from Aldrich Chemical Co. Lead(II) perchlorate hydrate was purchased from Aldrich Chemical Co. with purity over 99.0% and were used as received. Bis(triphenylphosphine)palladium(II) dichloride[1] and [(C^N^N)PtCl][2] [(C^N^N) = 4,4'-di-*tert*-butyl-6-phenyl-2,2'-bipyridine] were synthesized following reported procedures.

Physical Measurements and Instrumentation. All solvents were dried and purified by standard procedures. NMR spectra were recorded on Bruker, AV 400 spectrometers. ^{1}H and ^{13}C chemical shifts are determined by reference to residual ^{1}H and ^{13}C solvent signals. High-resolution mass spectra (HRMS) and Elemental Analysis were performed at the CRMPO (Centre de Mesures Physiques de l'Ouest) in Rennes. Absorption spectra were taken on Varian Cary 60 UV–vis absorption spectrophotometer. Fluorescence spectra were recorded on FS5 fluorescence spectrometer from Edinburgh Instrument™. Transient absorption and time related emission spectra were recorded on LP 920 Laser flash photolysis spectrometer from Edinburgh Instrument™. Quartz cuvettes (path-length = 1 cm) was used in all spectrophotometric and fluorometric measurements.

Synthesis

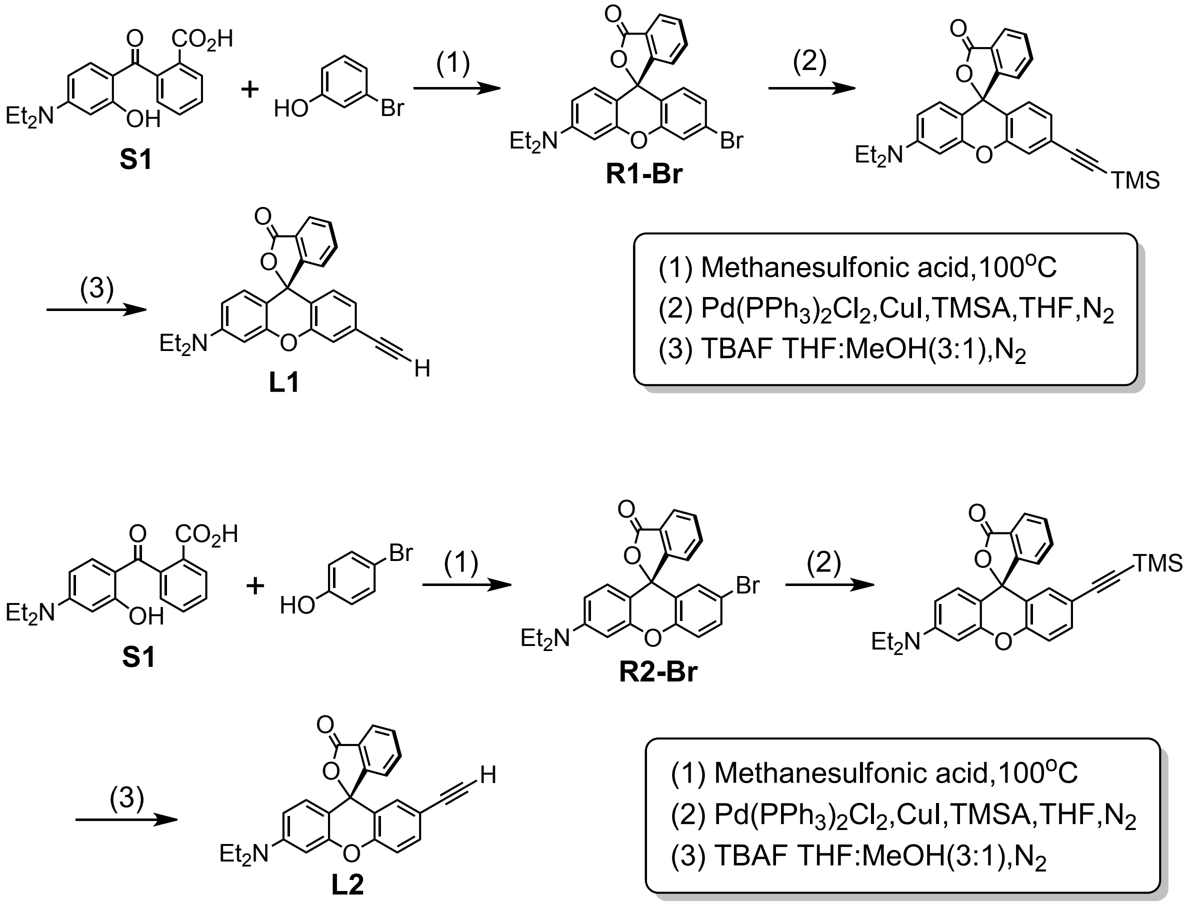

Scheme S1. The synthetic routes of **L1** and **L2**.

Synthesis of R1-Br. S1 (3.13g, 10 mmol) and 3-bromophenol (2.17 mL, 20 mmol) was added into methanesulfonic acid (30 mL) and heated at 100 °C overnight under stirring. Na_2CO_3 (aq) was used to tune the pH to about 7. Diluted with water and DCM, the organic layer of the resulting suspension was collected and dried over anhydrous $MgSO_4$. The crude product was purified by column chromatography with petroleum ether/ethyl acetate 4:1 as an eluting agent to afford **R1-Br** (2.8g, 62%). ^{1}H NMR (400 MHz, $CDCl_3$) δ 8.05 – 7.98 (m, 1H), 7.67 (td, *J* = 7.4, 1.3 Hz, 1H), 7.61 (td, *J* = 7.4, 1.1 Hz, 1H), 7.44 (d, *J* = 1.9 Hz, 1H), 7.20 – 7.15 (m, 1H), 7.11 (dd, *J* = 8.5, 2.0 Hz, 1H), 6.64 (d, *J* = 8.5 Hz, 1H), 6.57 (d, *J* = 8.9 Hz, 1H), 6.44 (d, *J* = 2.6 Hz, 1H), 6.37 (dd, *J* = 8.9, 2.6 Hz, 1H), 3.36 (q, *J* = 7.1 Hz, 4H), 1.18 (t, *J* = 7.1 Hz, 6H).

Synthesis of R2-Br. The procedure is similar to that described for the preparation of **R1-Br**, except 4-bromophenol was used in place of 3-bromophenol to give **R2-Br**. (3.2 g, 71%). ^{1}H NMR (400 MHz, $CDCl_3$) δ 8.03 (d, *J* = 7.4 Hz, 1H), 7.69 (td, *J* = 7.4, 1.3 Hz, 1H), 7.64 (dt, *J* = 7.4, 3.8 Hz, 1H), 7.46 (dd, *J* = 8.8, 2.4 Hz, 1H), 7.19 (d, *J* = 7.5 Hz, 1H), 7.15 (d, *J* = 8.8 Hz, 1H), 6.86 (d, *J* = 2.3 Hz, 1H), 6.56 (d, *J* = 8.9 Hz, 1H), 6.45 (d, *J* = 2.6 Hz, 1H), 6.37 (dd, *J* = 8.9, 2.6 Hz, 1H), 3.36 (q, *J* = 7.1 Hz, 4H), 1.17 (t, *J* = 7.1 Hz, 6H).

Synthesis of L1. **R1-Br** (450 mg, 1.0 mmol), $Pd(PPh_3)_2Cl_2$ (281 mg, 0.4 mmol), CuI (150 mg, 0.8 mmol), and TMSA (1.42 mL, 10 mmol) was dissolved in THF/Et_3N = 3:1 in N_2 atmosphere. The resulting mixture was heated to reflux overnight, then diluted with water and extracted with DCM. The organic layer was collected and dried over anhydrous MgSO4 and the solvent was removed. The remaining solid was then dissolved in 30 mL THF, and THF solution of TBAF (1.2 mL, 1 mol/L, 1.2 mmol) was added under N_2 atmosphere over ice-salt bath in the dark. The residue was purified by column chromatography with 9:1 petroleum ether / ethyl acetate as an eluent. Removal of the solvent afforded crude product as white powder. Sequent recrystallization by slowly evaporation of solution in DCM: hexane = 20:1 provided the target compound (162 mg, 41%). ^{1}H NMR (400 MHz, $CDCl_3$) δ 8.04 (d, *J* = 7.4 Hz, 1H), 7.66 (dt, *J* = 14.7, 6.9 Hz, 2H), 7.41 (s, 1H), 7.20 (d, *J* = 7.5 Hz, 1H), 7.11 (dd, *J* = 8.2, 1.4 Hz, 1H), 6.75 (d, *J* = 8.1 Hz, 1H), 6.60 (d, *J* = 8.9 Hz, 1H), 6.48 (d, *J* = 2.5 Hz, 1H), 6.39 (dd, *J* = 8.9, 2.5 Hz, 1H), 3.39 (q, *J* = 7.0 Hz, 4H), 3.14 (s, 1H), 1.20 (t, *J* = 7.0 Hz, 6H).

Synthesis of L2. The procedure is similar to that described for the preparation of **L1**, except **R2-Br** was used in place of **R1-Br** to give **L2** (118 mg, 30%). ^{1}H NMR (400 MHz, $CDCl_3$) δ 8.05 (d, *J* = 7.3 Hz, 1H), 7.74 – 7.60 (m, 2H), 7.50 (dd, *J* = 8.6, 2.0 Hz, 1H), 7.25 – 7.17 (m, 2H), 6.94 (d, *J* = 1.9 Hz, 1H), 6.59 (d, *J* = 8.9 Hz, 1H), 6.48 (d, *J* = 2.5 Hz, 1H), 6.39 (dd, *J* = 8.9, 2.6 Hz, 1H), 3.39 (q, *J* = 7.1 Hz, 4H), 2.97 (s, 1H), 1.20 (t, *J* = 7.1 Hz, 6H).

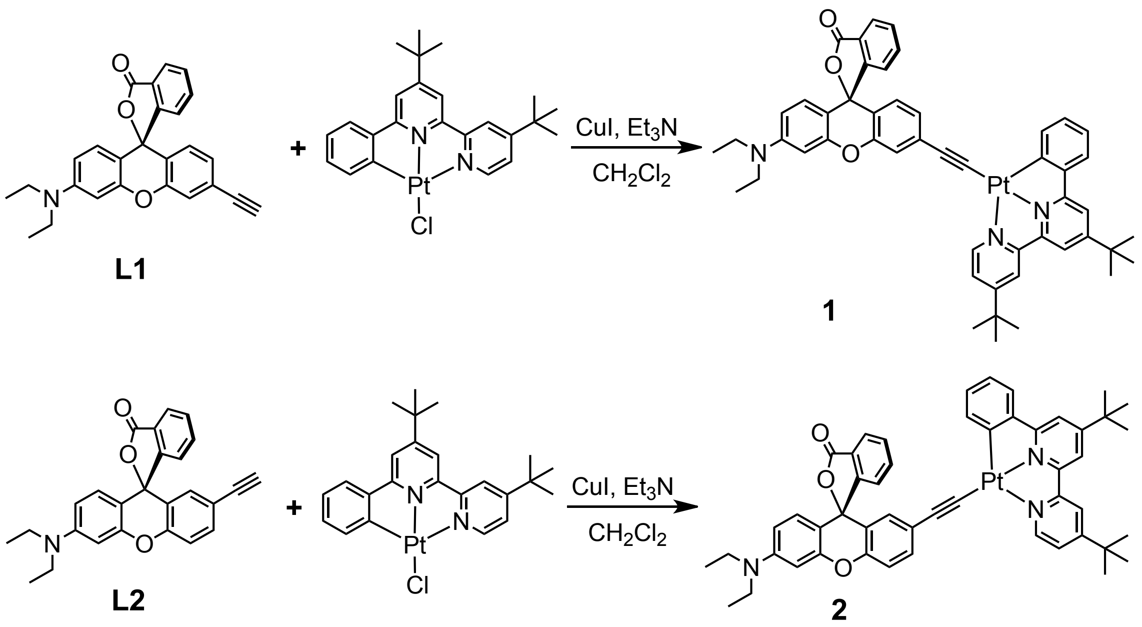

Scheme S2. The synthetic routes of **1** and **2**.

Procedure for synthesis of complexes 1 and 2. To a dry and degassed $CH_2Cl_2/{}^iPr_2NH$ (4/1: 20 mL) solution of 4,4'-di(*tert*-butyl)-6-phenyl-2,2'-bipyridine platinum(II) chloride [(C^N^N)PtCl)] (0.217 g, 0.38 mmol), **L1** or **L2** (0.145 g, 0.38 mmol) and CuI (0.001 g, 0.04 mmol) were added. After 15 h of stirring at 30ºC in the dark, the solvents were removed under reduce pressure. The residue was purified by column chromatography (SiO_2, CH_2Cl_2 → CH_2Cl_2/AcOEt: 9/1) to afford **1** or **2** (70 % brown powder).

1: ^{1}H NMR (400 MHz, $CDCl_3$): δ = 9.00 (d, J = 4 Hz, 1H, N^N^C), 8.00 (d, J = 8 Hz, 1H, Rhod.), 7.89 (d, J = 4 Hz, 1H, N^N^C), 7.79 (d, J = 8 Hz, 1H, N^N^C), 7.71 (t, J = 8 Hz, 1H, N^N^C), 7.68 – 7.60 (m, 3H, N^N^C), 7.55 (dd, ^1J = 8 Hz, ^2J = 4 Hz, 1H, Rhod.), 7.45 (d, J = 8 Hz, 1H, Rhod.), 7.37 (d, J = 4 Hz, 1H, Rhod.), 7.23 (d, J = 8 Hz, 1H, Rhod.), 7.18 – 7.05 (m, 1H Rhod.+ 2H N^N^C), 6.66 (d, J = 8 Hz, 1H, Rhod.), 6.59 (s, 1H, Rhod.), 6.57 (s, 1H, Rhod.), 6.39 (dd, ^1J = 8 Hz, ^2J = 4 Hz, 1H, Rhod.), 3.39 (q, J = 8 Hz, 4H, Rhod.), 1.45 (s, 9H, N^N^C), 1.43 (s, 9H, N^N^C), 1.20 (t,, J = 8 Hz, 6H, Rhod). ^{13}C [^{1}H] NMR (100 MHz, CD_2Cl_2): δ = 169.9, 165.3, 164.5, 164.4, 158.6, 155.1, 153.7, 153.4, 151.8, 150.2, 149.9, 147.9, 143.0, 138.7, 135.5, 134.4, 131.5, 131.0, 130.1, 129.3, 127.8, 125.3, 125.3, 124.9, 124.7, 124.6,

124.0, 120.1, 119.7, 117.1, 116.3, 115.5, 108.9, 106.9, 105.7, 104.7, 98.1, 84.4, 45.0, 36.5, 36.2, 30.7, 30.6, 30.5, 12.8. HRMS: *m/z* $[M+H]^+$ Calcd. for $C_{50}H_{48}N_3O_3{}^{195}Pt$ 933.3338, found: 933.3338. Elemental Analysis (%): Calcd. for $C_{50}H_{47}N_3O_3Pt{\bullet}1/4CH_2Cl_2$: C, 63.25; H, 5.02; N, 4.40. Found: C, 63.25; H, 4.78; N, 4.41.

2: ^{1}H NMR (400 MHz, $CDCl_3$): δ = 8.94 (d, *J* = 4 Hz, 1H, N^N^C), 7.98 (d, *J* = 8 Hz, 1H, Rhod.), 7.87 (d, *J* = 4 Hz, 1H, N^N^C), 7.73 – 7.60 (m, 5H, N^N^C), 7.54 (dd, ^1J = 8 Hz, ^2J = 4 Hz, 1H, Rhod.), 7.52 (dd, ^1J = 8 Hz, ^2J = 4 Hz, 1H, Rhod.), 7.45 (dd, ^1J = 8 Hz, ^2J = 4 Hz, 1H, Rhod.), 7.23 (d, *J* = 8 Hz, 1H, Rhod.), 7.18 (d, *J* = 8 Hz, 1H, Rhod.), 7.10 (td, ^1J = 8 Hz, ^2J = 4 Hz, 1H, N^N^C), 7.05 (td, ^1J = 8 Hz, ^2J = 4 Hz, 1H, N^N^C), 6.88 (d, *J* = 4 Hz, 1H, Rhod.), 6.58 (s, 1H, Rhod.), 6.55 (s, 1H, Rhod.), 6.49 (d, *J* = 4 Hz, 1H, Rhod.), 6.38 (dd, ^1J = 8 Hz, ^2J = 4 Hz, 1H, Rhod.), 3.38 (q, *J* = 8 Hz, 4H, Rhod.), 1.44 (s, 9H, N^N^C), 1.42 (s, 9H, N^N^C), 1.18 (t, *J* = 8 Hz, 6H, Rhod). ^{13}C [^{1}H] NMR (100 MHz, CD_2Cl_2): δ = 169.4, 164.7, 163.9, 163.8, 158.0, 154.5, 153.1, 152.8, 151.3, 149.7, 149.3, 147.4, 142.4, 138.2, 134.8, 133.8, 130.9, 130.4, 129.5, 128.7, 127.2, 124.8, 124.7, 124.4, 124.1, 124.0, 123.4, 119.5, 119.1, 116.5, 115.7, 114.9, 108.3, 106.3, 105.0, 104.1, 97.5, 83.8, 44.4, 35.9, 35.6, 30.2, 30.0, 12.3. HRMS: *m/z* $[M+H]^+$ Calcd. For $C_{50}H_{48}N_3O_3{}^{195}Pt$ 933.3338, found: 933.3336. Elemental Analysis (%): Calcd. for $C_{50}H_{47}N_3O_3Pt{\bullet}½CH_2Cl_2$: C, 62.18; H, 4.96; N, 4.31 Found: C, 62.81; H, 5.06; N, 4.43.

Singlet Oxygen Quantum Yield Determination

Singlet oxygen emission was detected by using FLS-980 spectrofluorometer. All of the compounds were dissolved in CH_3CN. The absorbance at 514.5 nm, as the excitation wavelength, was adjusted to be around 0.36 for opened forms of **1** and **2**; and Rose Bengal. Upon measurements, an 850 nm long-pass filter was inserted in between the sample and the detector to avoid any high-order diffraction from the visible emission. Singlet oxygen quantum yield was determined by comparing the $^1O_2$ emission intensity of Rose Bengal (Φ_Δ = 45% in CH_3CN).

Computational Details

All geometries and energies presented in this study were computed using the B3PW91 density functional theory method as implemented in the Gaussian 09 program package.[3] Geometry optimizations were performed using basis set 6-31++G*, Meanwhile, LANL2DZ effective-core potentials are used for heavy atom (Pt). The stationary structures are obtained by verifying that all of the harmonic frequencies are real.

X-ray crystallography

Single crystals of **1** and **2** were grown by slow diffusion of diethyl ether vapors into a saturated dichloromethane solution of the corresponding complex. The unit cell and data collection summarized in Table S1 and S2. The structures were solved by dual-space algorithm using the *SHELXT* program,[4] and then refined with full-matrix least-square methods based on F^2 (*SHELXL-2014*).[5] The contribution of the disordered solvents to the calculated structure factors was estimated following the *BYPASS* algorithm,[6] implemented as the *SQUEEZE* option in *PLATON*.[7] A new data set, free of solvent contribution, was then used in the final refinement. All non-hydrogen atoms were refined with anisotropic atomic displacement parameters. H atoms were finally included in their calculated positions.

Table S1. Crystal structure determination of 1. CCDC 1503542

An orange crystal of dimensions 0.58 mm x 0.15 mm x 0.09 mm mounted in a glass capillary was used for data collection:

Empirical formula	$C_{51}H_{49}Cl_2N_3O_3Pt$
Extended formula	$C_{50}H_{47}N_3O_3Pt{\bullet}CH_2Cl_2$
Formula weight	1017.92
Temperature	150 *K*
Wavelength	0.71073 Å
Crystal system, space group	monoclinic, $P2_1/c$
Unit cell dimensions	a = 11.4845(19) Å, α = 90°
	b = 24.408(5) Å, β = 91.637(7)°
	c = 17.785(4) Å, γ = 90°
Volume	4983.4(16) $Å^3$
Z, Calculated density	4, 1.357 ($g.cm^{-3}$)
Absorption coefficient	2.964 mm^{-1}
F(000)	2048
Crystal size	0.580 x 0.150 x 0.090 mm
Crystal color	yellow
Theta range for data collection	2.938 to 27.483°
h_min, h_max	-14, 14
k_min, k_max	-23, 31
l_min, l_max	-23, 20
Reflections collected / unique	31653 / 11243 [R(int) = 0.0716]
Reflections [I>2σ]	7244
Completeness to theta_max	0.985
Absorption correction type	multi-scan
Max. and min. transmission	0.766, 0.430
Refinement method	Full-matrix least-squares on F^2
Data / restraints / parameters	11243 / 0 / 483
[b]Goodness-of-fit	1.080
Final *R* indices [I>2σ]	$R1^c$ = 0.0878, $wR2$ = 0.2174
R indices (all data)	$R1^c$ = 0.1347, $wR2$ = 0.2352
Largest diff. peak and hole	3.123 and -3.525 $e^-.Å^{-3}$

Table S2. Crystal structure determination of 2. CCDC 1507269

An orange crystal of dimensions 0.60 mm x 0.25 mm x 0.21 mm mounted in a glass capillary was used for data collection:

Empirical formula	$C_{50}H_{45}N_3O_3Pt$
Formula weight	930.98
Temperature	150(2) *K*
Wavelength	0.71073 Å
Crystal system, space group	triclinic, *P*-1
Unit cell dimensions	a = 9.0585(14) Å, α = 100.971(5)°
	b = 15.851(3) Å, β = 101.370(5)°
	c = 16.470(3) Å, γ = 104.503(5)°
Volume	2171.8(6) $Å^3$
Z, Calculated density	2, 1.424 ($g.cm^{-3}$)
Absorption coefficient	3.275 mm^{-1}
F(000)	936
Crystal size	0.600 x 0.250 x 0.210 mm
Crystal color	orange
Theta range for data collection	2.969 to 27.476°
h_min, h_max	-7, 11
k_min, k_max	-20, 20
l_min, l_max	-21, 21
Reflections collected / unique	50344 / 9875 [R(int) = 0.0534]
Reflections [I>2σ]	8137
Completeness to theta_max	0.992
Absorption correction type	multi-scan
Max. and min. Transmission	0.503 , 0.273
Refinement method	Full-matrix least-squares on F^2
Data / restraints / parameters	9875 / 11 / 499
[b]Goodness-of-fit	1.000
Final *R* indices [I>2σ]	$R1^c$ = 0.0490, *wR*2 = 0.1216
R indices (all data)	$R1^c$ = 0.0683, *wR*2 = 0.1402
Largest diff. peak and hole	3.848 and -3.066 $e^-.Å^{-3}$

Table S3. Calculated structural data of **1** and **2**.

	**1**			**2**		
	Bond distance in closed form (Å)	Bond distance in opened form (Å)	Δ distance = opened form – closed form (Å)	Bond distance in closed form (Å)	Bond distance in opened form (Å)	Δ distance = opened form – closed form (Å)
**C1-C2**	1.23	1.24	+0.01	1.23	1.23	0.00
**C2-C3**	1.42	1.40	-0.02	1.42	1.41	-0.01
**C1-Pt**	1.95	1.93	-0.02	1.94	1.94	0.00

Table S4. HOMO and LUMO compositions (%) for the the lowest triplet (T_1) state of opened forms of **1** and **2**

	opened form of **1**			opened form of **2**		
	Pt	C≡C	xanthene	Pt	C≡C	xanthene
LUMO	2.6	8.1	86.7	0.3	1.2	98.3
HOMO	21.1	25.6	32.5	18.3	39.3	34.7

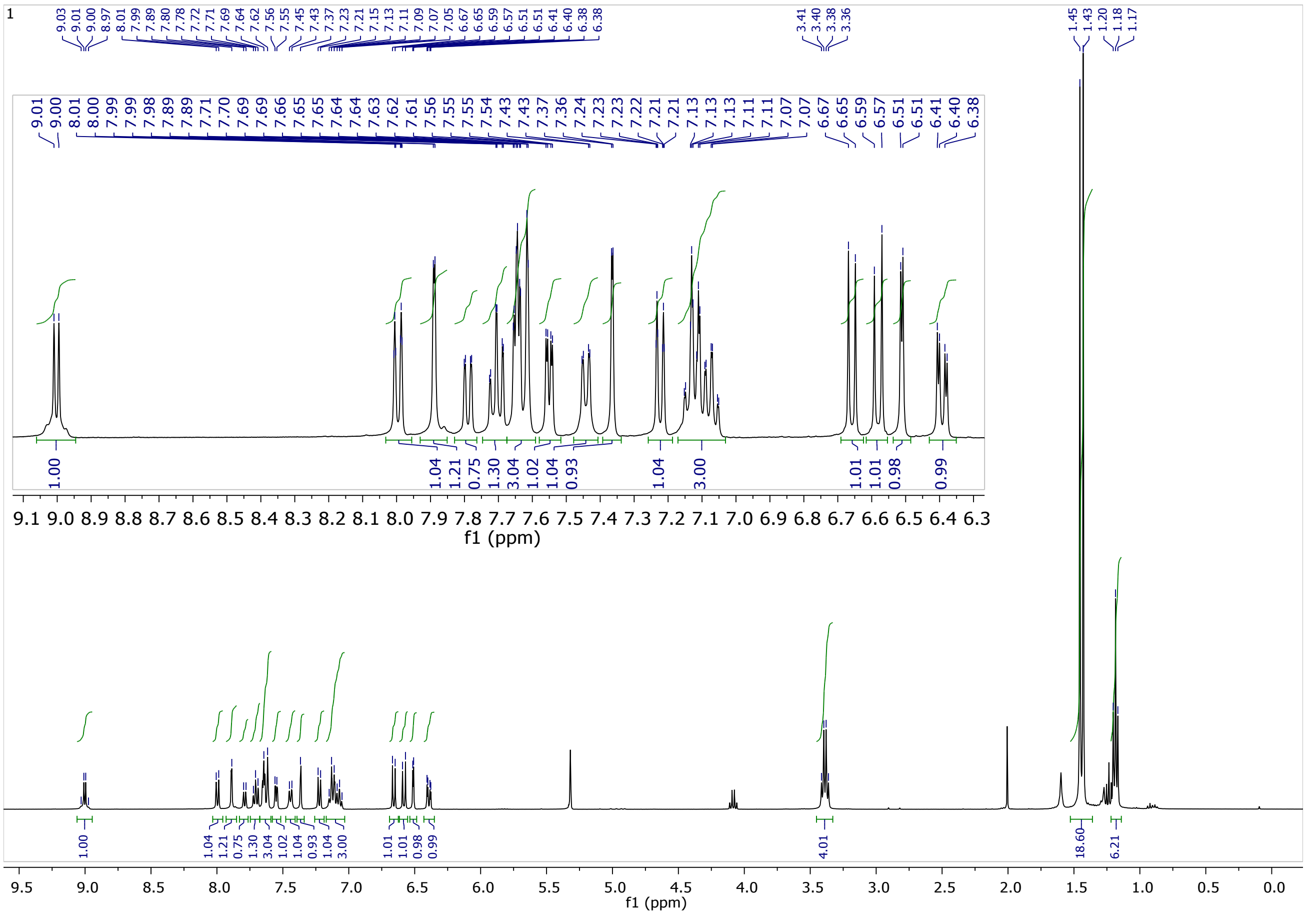

Figure S1. ^1H NMR spectrum of **1** in CD_2Cl_2.

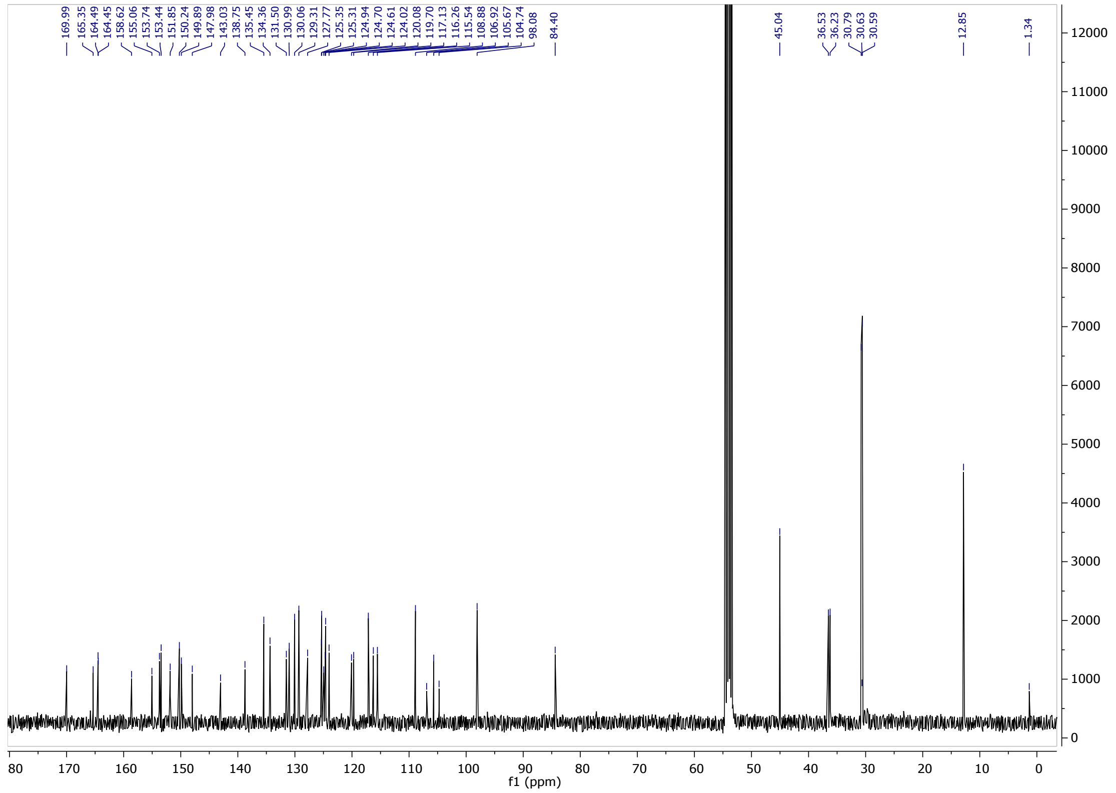

Figure S2. $^{13}C[^{1}H]$ NMR spectrum of **1** in CD_2Cl_2.

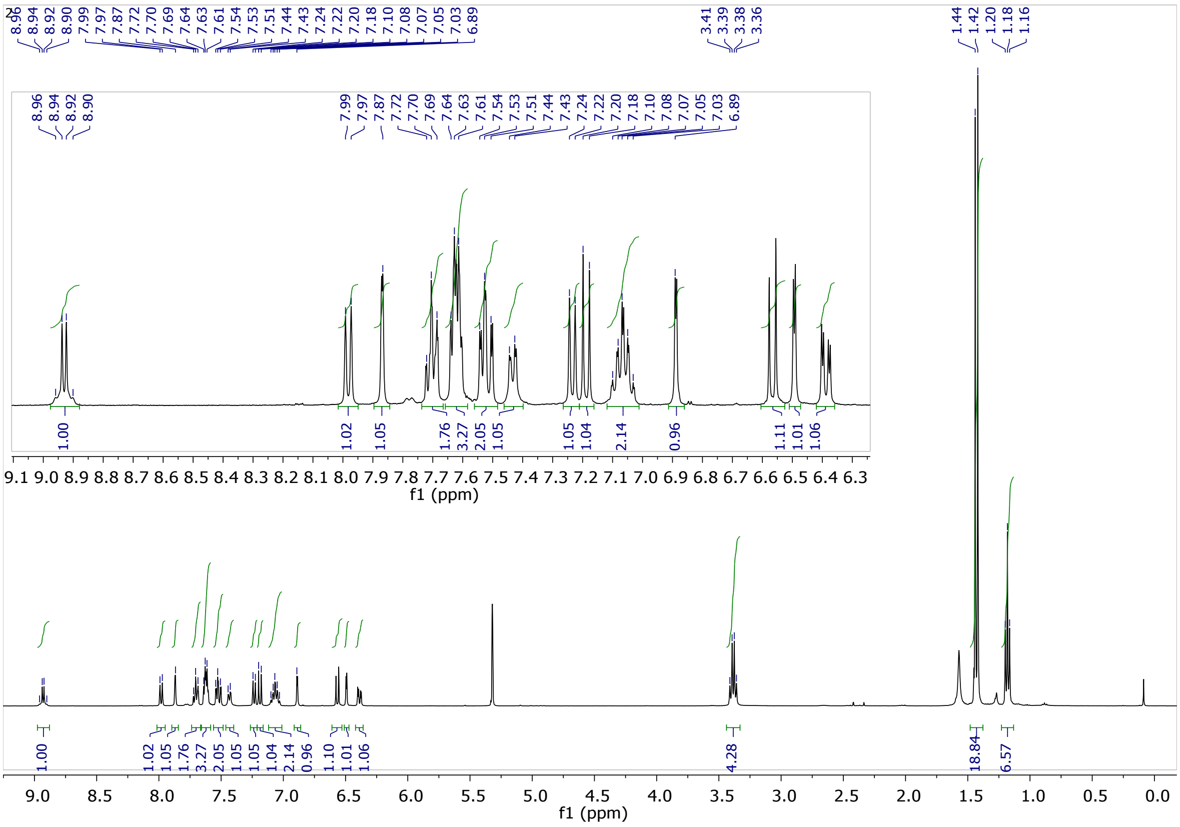

Figure S3. ^{1}H NMR spectrum of **2** in CD_2Cl_2.

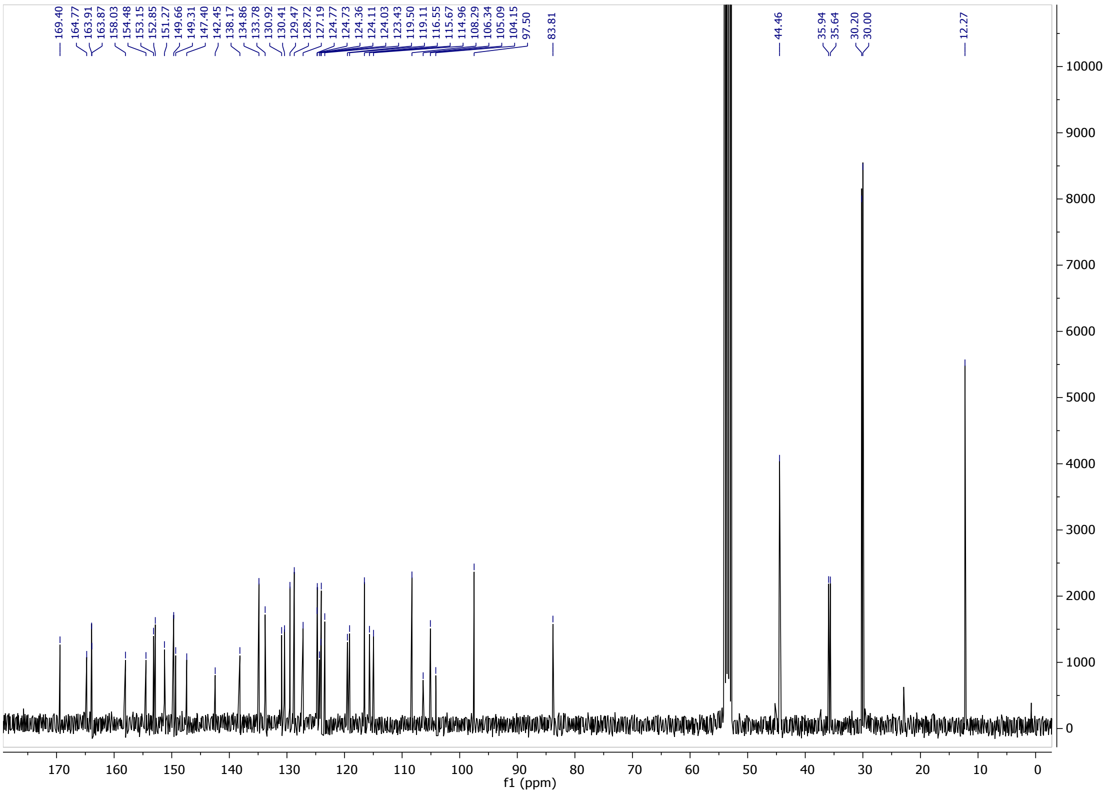

Figure S4. $^{13}C[^{1}H]$ NMR spectrum of **2** in CD_2Cl_2.

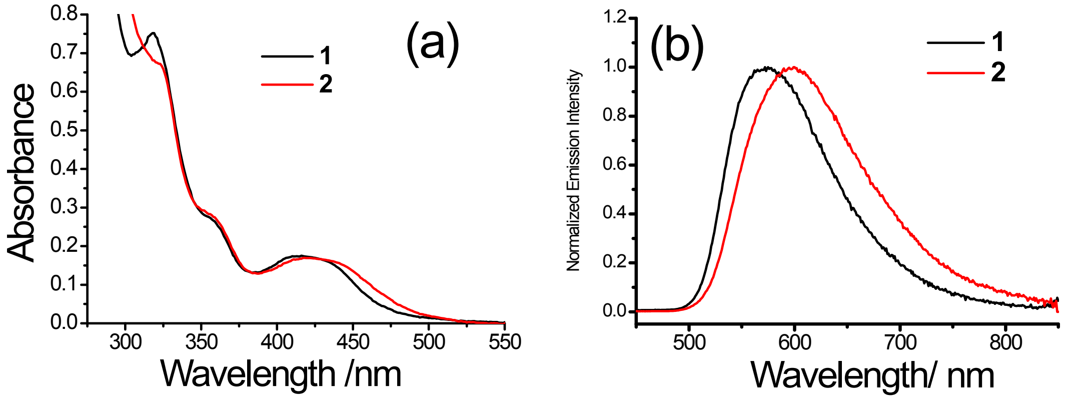

Figure S5. Electronic absorption (a) and emission (b) spectra of **1** and **2** in acetonitrile at room temperature. Deoxygenated samples were prepared for emission measurement.

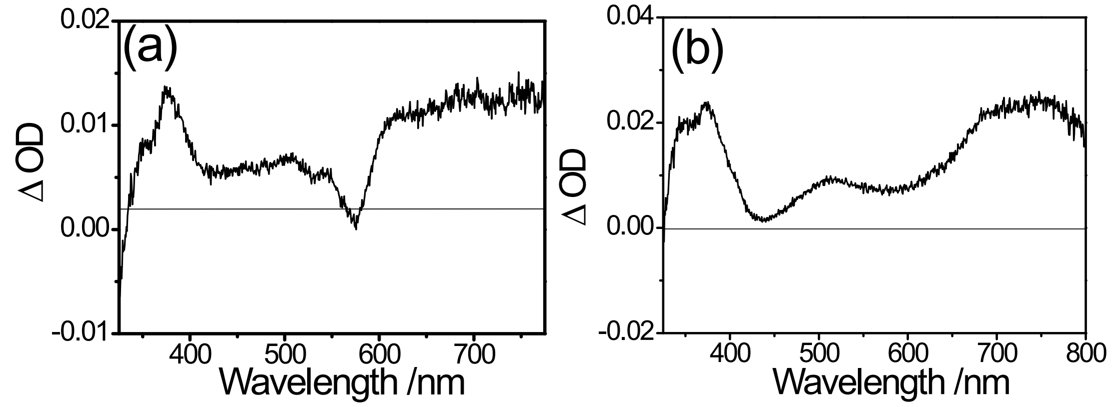

Figure S6. (a) Transient absorption different spectra at time zero of **1** (a) and **2** (b) in deoxygenated acetonitrile at room temperature following a 355-nm laser pulse excitation.

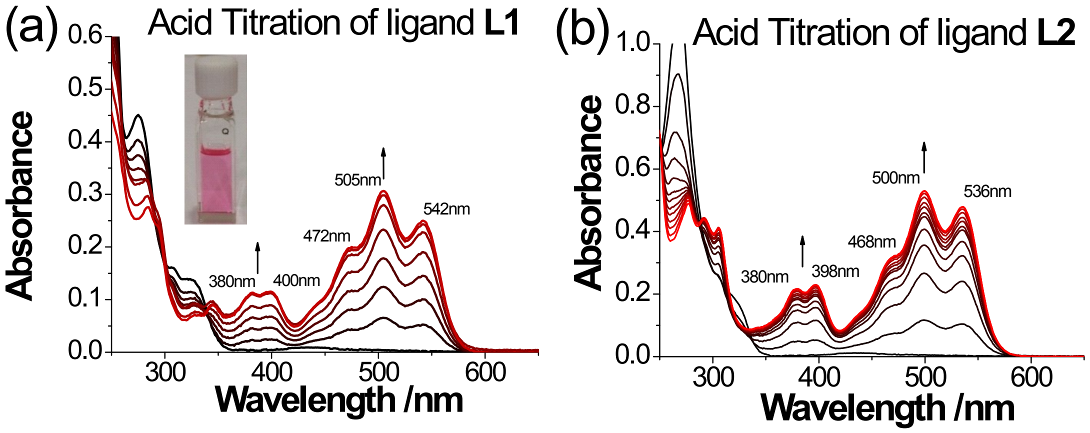

Figure S7. Electronic absorption spectral changes of (a) **L1** (conc. = 1E-5M) and (b) **L2** (conc. = 1E-5M) upon addition of various concentrations of proton in acetonitrile at room temperature.

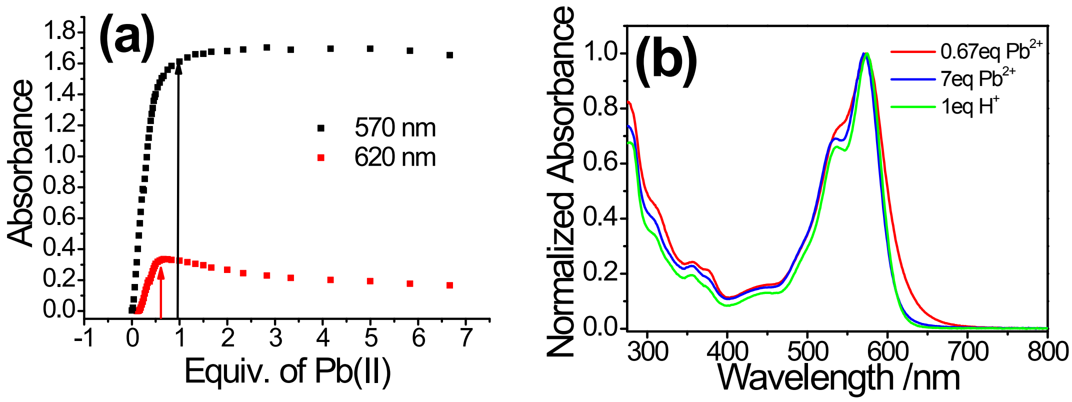

Figure S8. (a) The plot of absorbance at 570 nm and 620 nm as a function of equivalents of Pb(II) ion. (b) Normalized electronic absorption spectra of **1** in the presence of 0.67 and 7.0 equivalents of Pb(II) ion; and 1.0 equivalent of acid in acetonitrile.

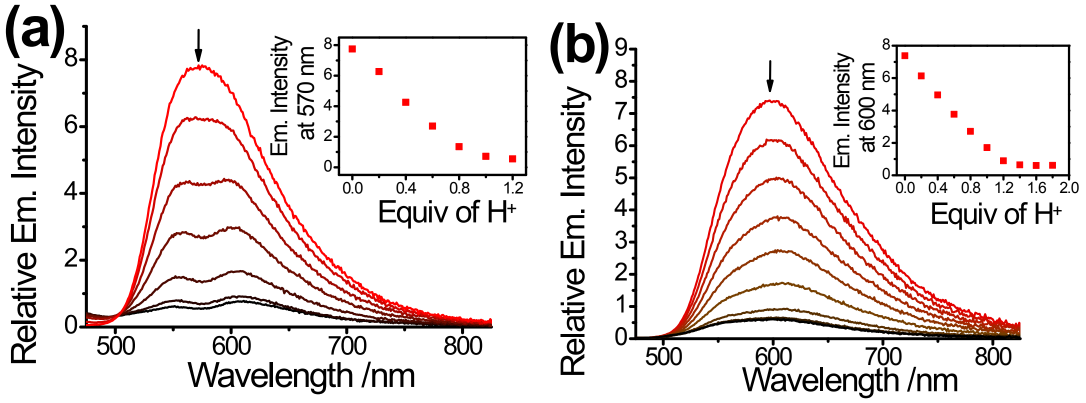

Figure S9. Emission spectral changes of (a) **1** (conc. = 1E-5M) and (b) **2** (conc. = 1E-5M) upon addition of various equivalents of proton in air-saturated acetonitrile with the excitation wavelength at 348 nm for **1** and 330 nm for **2** at room temperature.

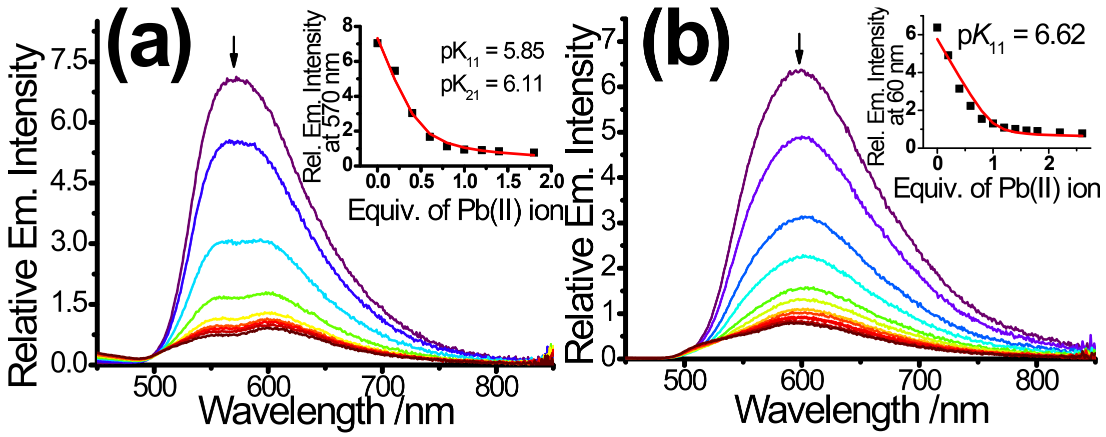

Figure S10. Emission spectral changes of (a) **1** and (b) **2** (conc. = 10μM) with various concentrations of Pb(II) ion in air-saturated acetonitrile with the excitation wavelength at 348 nm for **1** and 330 nm for **2**. Insets show the plot of relative emission intensity versus the equivalents of Pb(II) ion and the theoretical fitting curves.

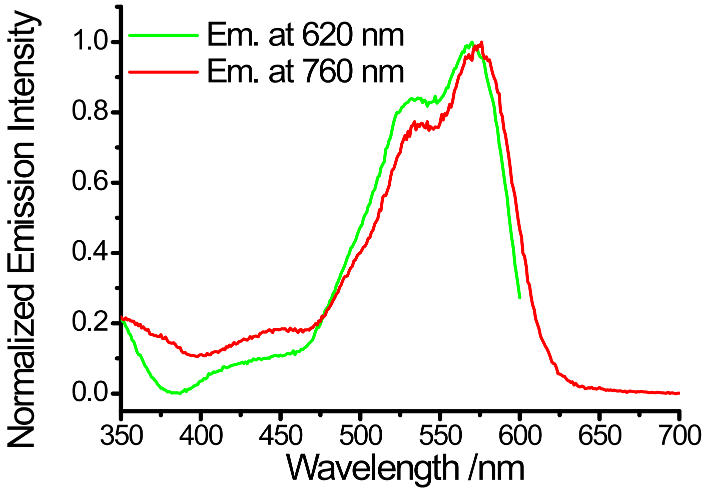

Figure S11. Excitation spectra of opened form of **1** monitored at 620 nm and 760 nm in its ring-opened form triggered by Pb(II) ion in acetonitrile.

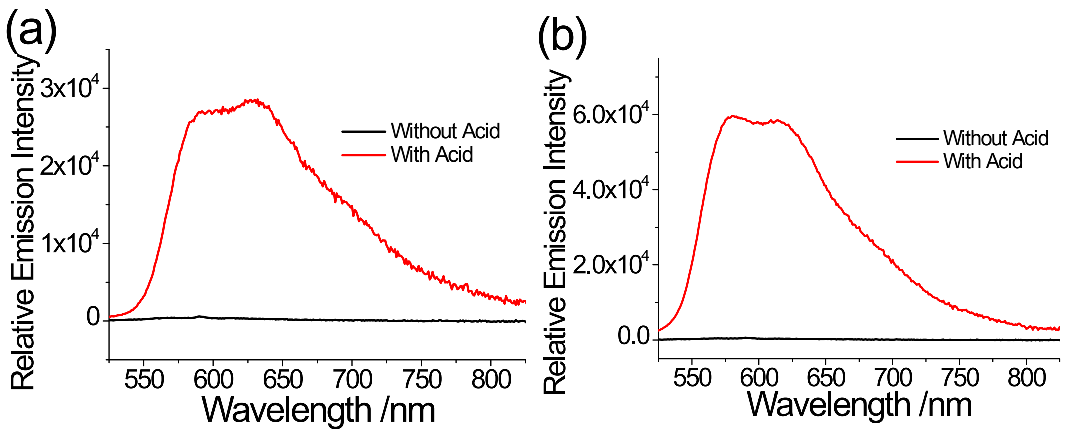

Figure S12. Emission spectral changes of (a) **L1** and (b) **L2** upon addition of various concentrations of proton in air-saturated acetonitrile at room temperature.

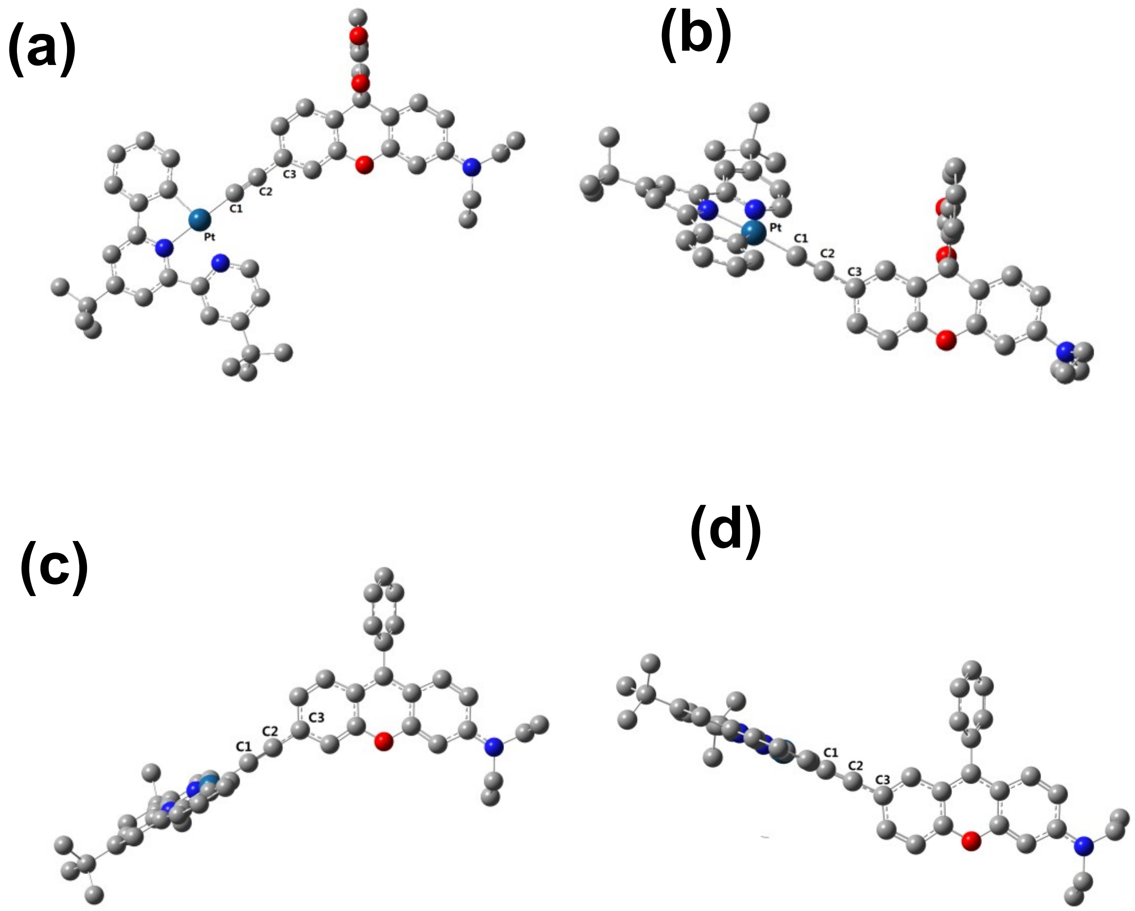

Figure S13. Optimized structure of ring-closed form of **1** (a) and **2** (b); ring-opened form of **1** (c) and **2** (d).

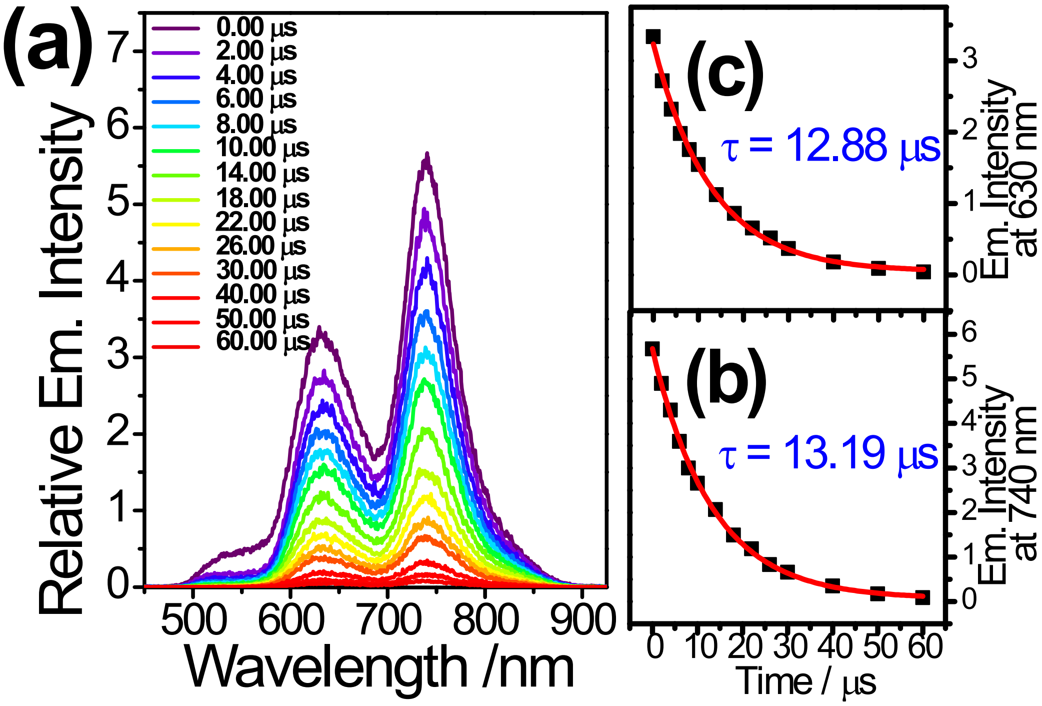

Figure S14. (a) Time-resolved emission spectra of **1** in the ring-opened form in deoxygenated dichloromethane (with 1% acetonitrile) at room temperature and the corresponding lifetimes decay traces monitored at (b) 740 nm (with exclusion of the first data point) and (c) 620nm. Pulsed laser at excitation wavelength of 532 nm.

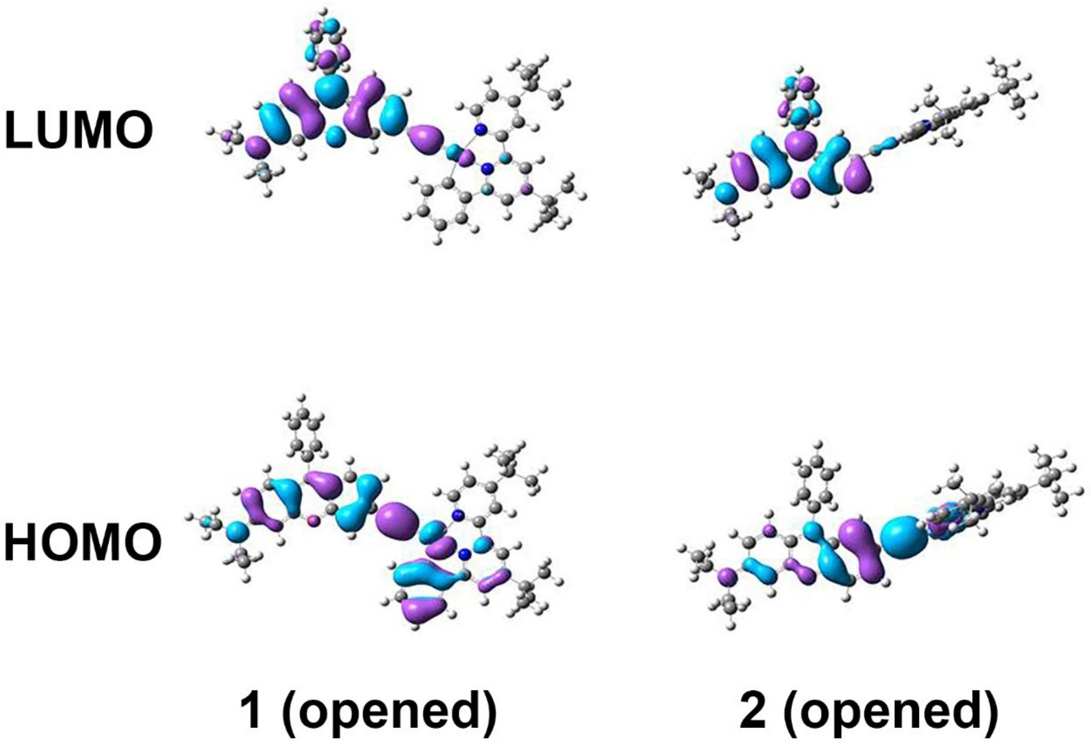

Figure S15. The electronic density distribution of the HOMO and LUMO for the the lowest triplet (T_1) state of opened forms of **1** and **2**.

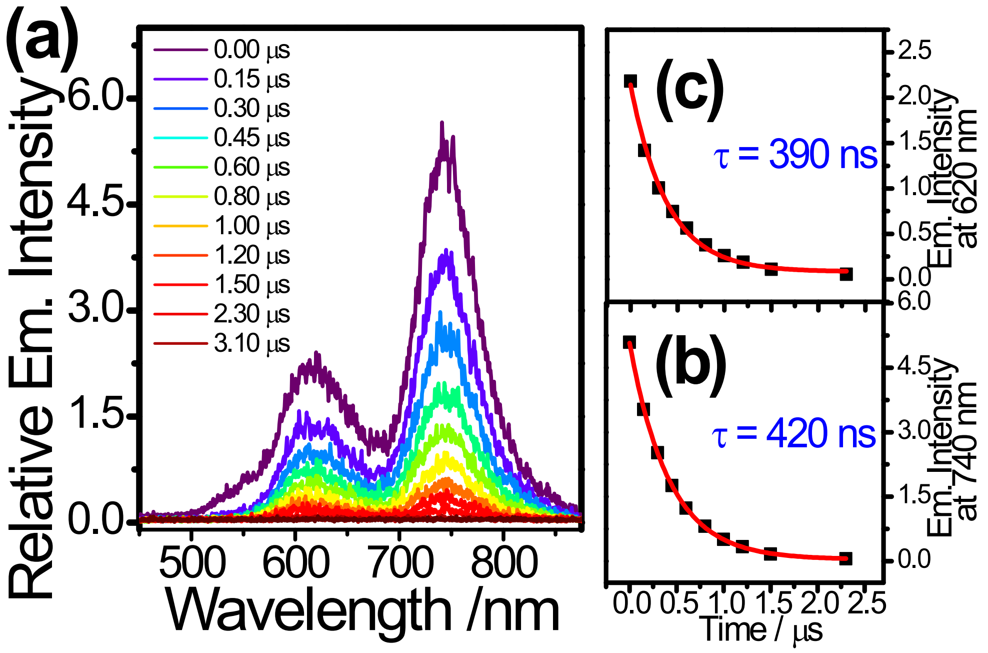

Figure S16. (a) Time-resolved emission spectra of **1** in the ring-opened form in air-saturated acetonitrile at room temperature and the corresponding lifetimes decay traces monitored at (b) 740 nm (with exclusion of the first data point) and (c) 620nm. Pulsed laser at excitation wavelength of 532 nm.

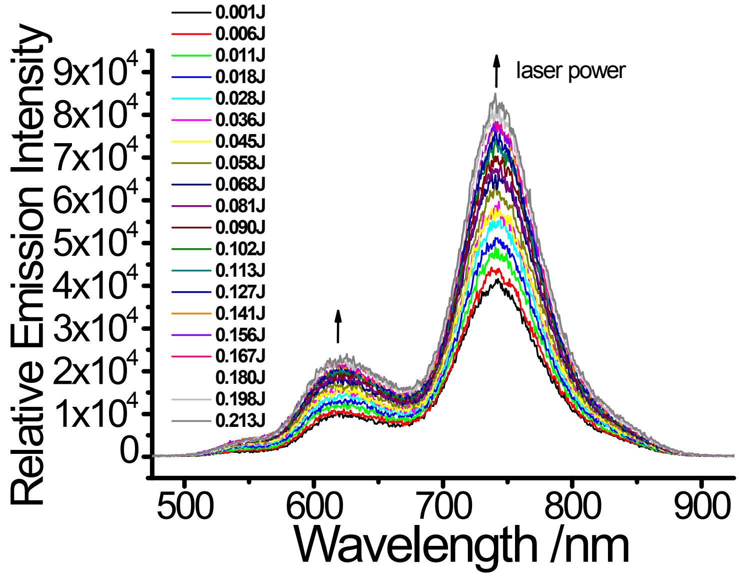

Figure S17. (a) Emission spectra of **1** in the ring-opened form in deoxygenated acetonitrile at room temperature with different excitation power intensity.

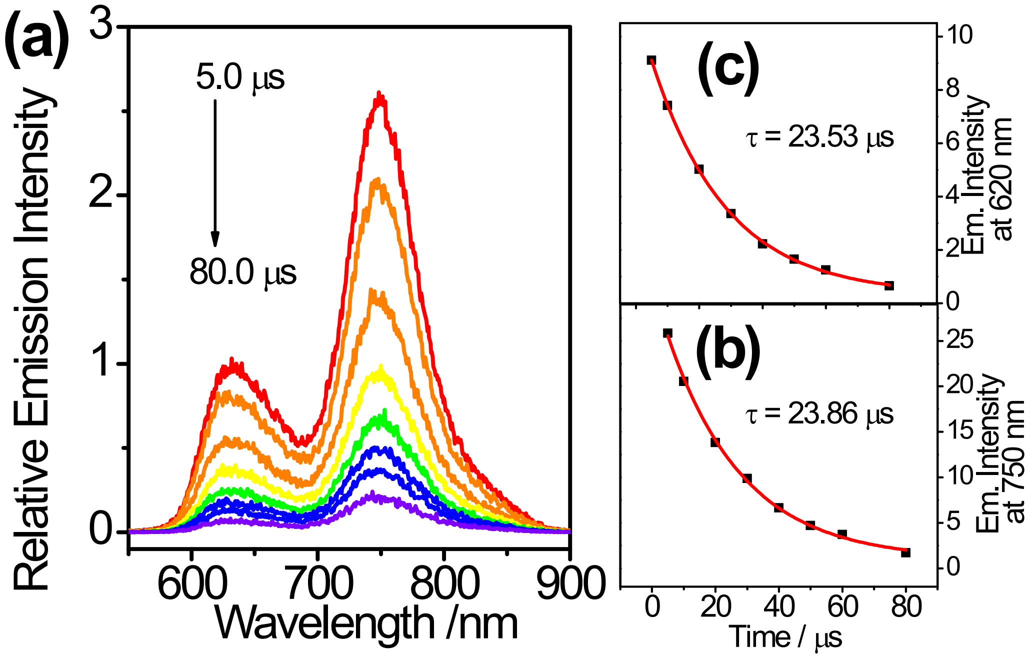

Figure S18. (a) Time-resolved emission spectra of **1** in the ring-opened form in deoxygenated glycerol triacetate at room temperature and the corresponding lifetimes decay traces monitored at (b) 740 nm and (c) 620nm. Pulsed laser at excitation wavelength of 532 nm.

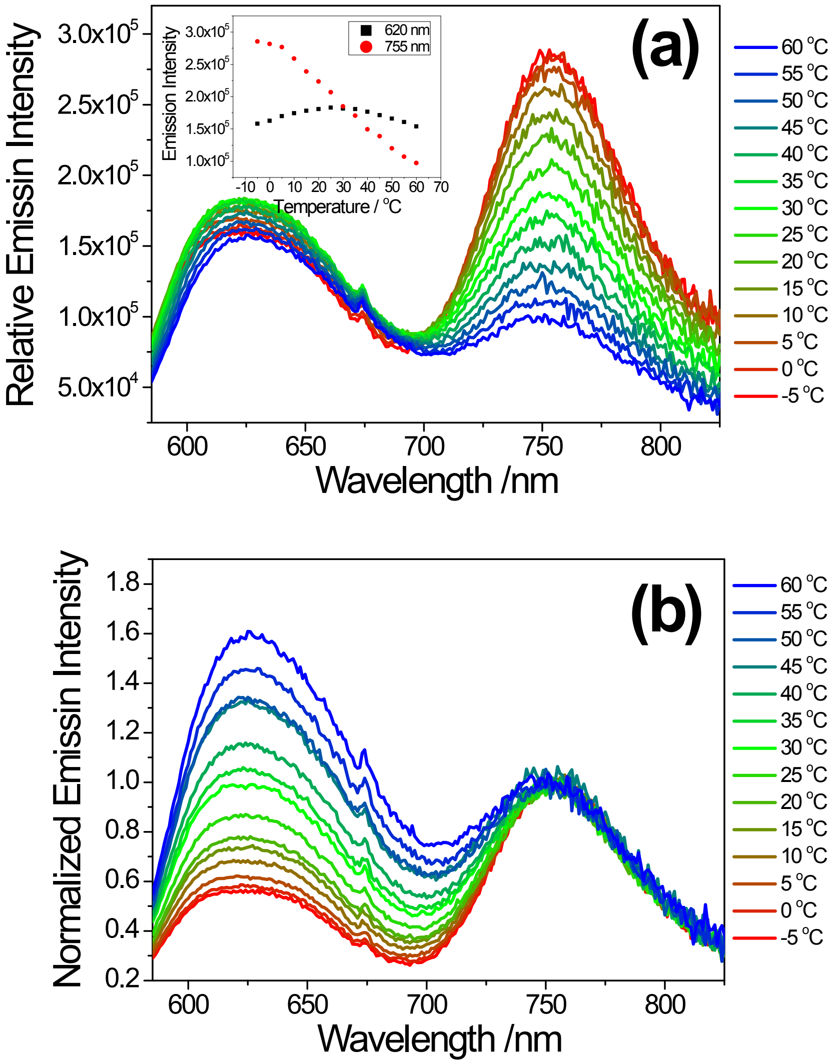

Figure S19. Variable-temperature emission spectra of **1** in the ring-opened form in deoxygenated acetonitrile (a) without normalization and (b) with normalization at 750 nm.

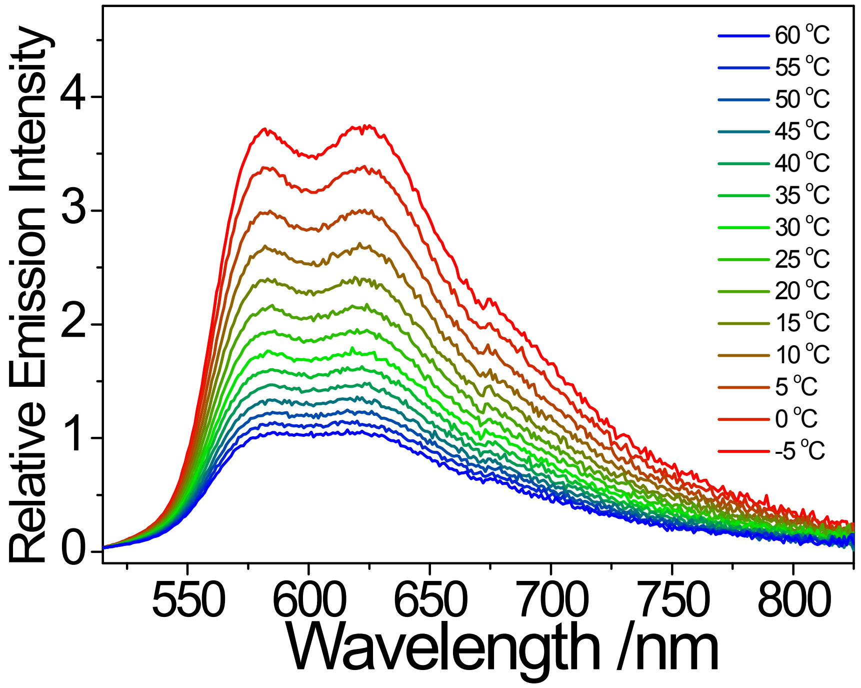

Figure S20. Variable-temperature emission spectra of **L1** in the ring-opened form in deoxygenated acetonitrile.

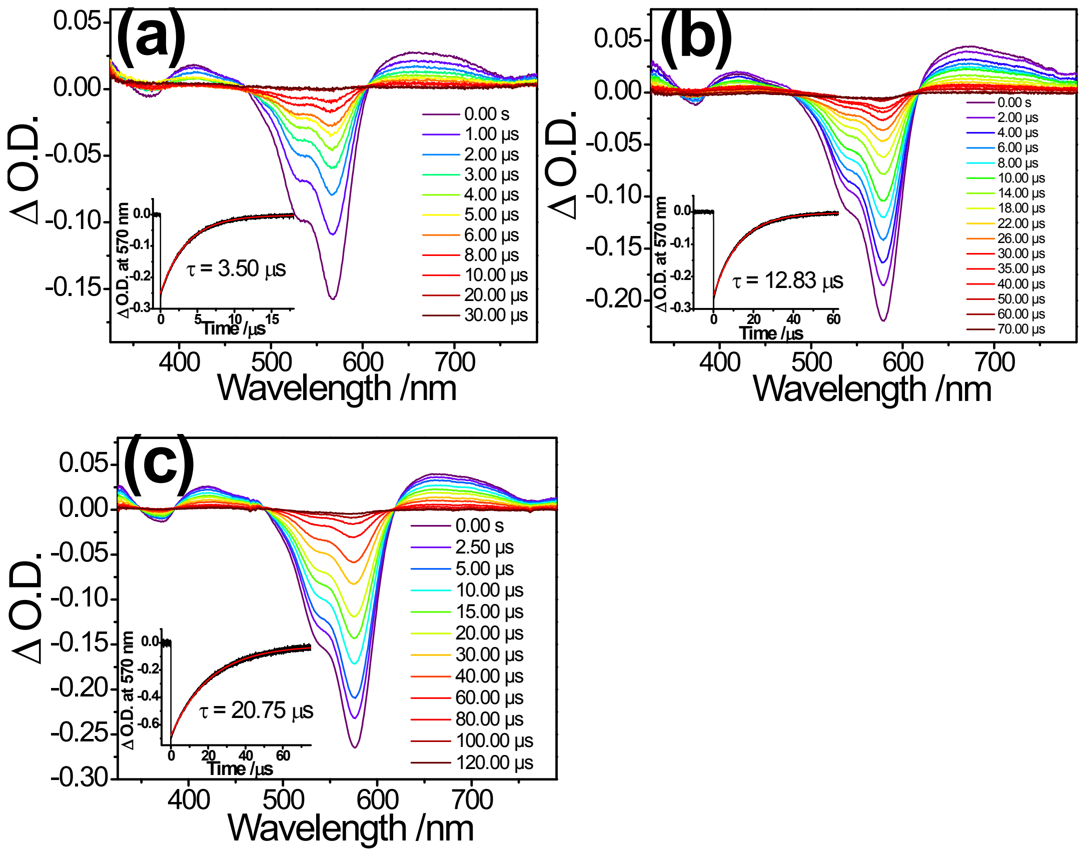

Figure S21. Transient absorption different spectral change of **1** (conc. = 10μM) (conc. = 20μM) in their ring-opened forms with excess amount of Pb(II) ion in deoxygenated acetonitrile (a), deoxygenated dichloromethane (with 1% acetonitrile) (b) and deoxygenated glycerol triacetate at room temperature. Insets show the transient absorption lifetime fitting of the signal monitoring at 570 nm. Excitation wavelength = 532 nm.

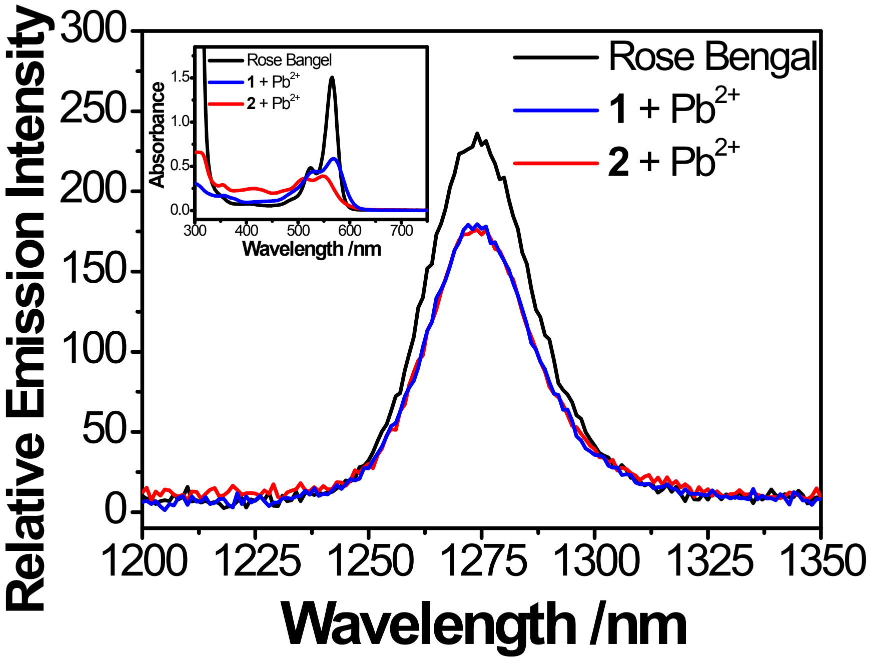

Figure S22. Singlet oxygen emission spectra of **1** and **2** with excess amount of Pb(II) ion and Rose Bengal. Inset shows their corresponding absorption spectra in the measurement.

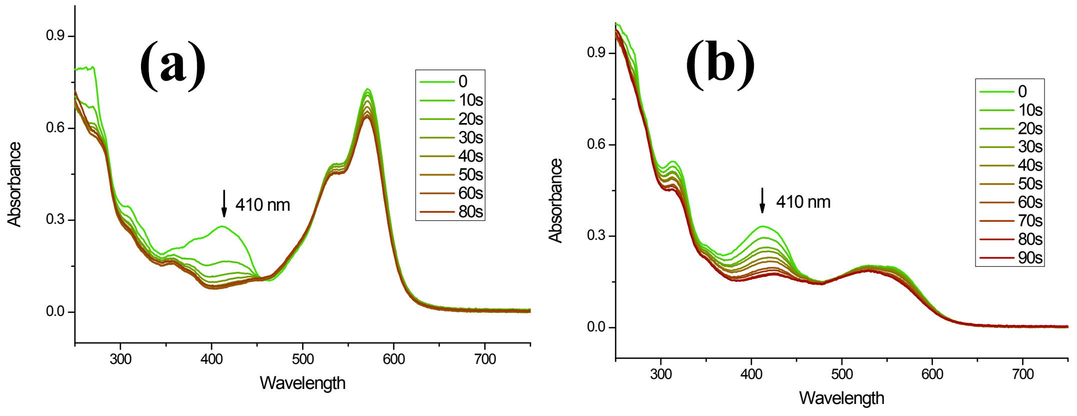

Figure S23. Singlet oxygen generation measurement based on the absorption decay of DBPF for **1** (a) and **2** (b).

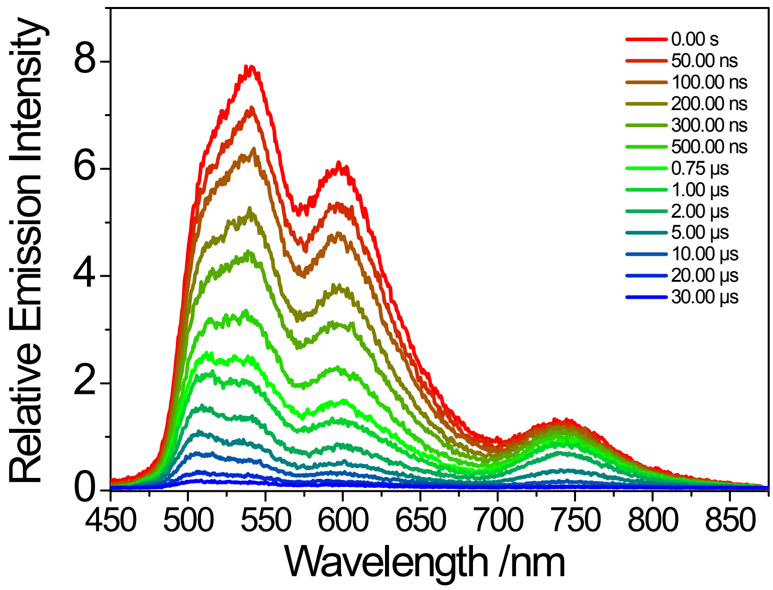

Figure S24. Time-resolved emission spectra of **1** in the ring-opened form in deoxygenated acetonitrile at room temperature with 355 nm pulse laser excitation.

References

(1) N. Miyaura, A. Suzuki, *Org. Synth.* 1990, **68**, 130.

(2) W. Lu, W.-M. Kwok, C. Ma, C. T.-L. Chan, M.-X. Zhu, C.-M. Che, *J. Am. Chem. Soc.* 2011, **133**, 14120-14135.

(3) M.J. Frisch, G.W. Trucks, H.B. Schlegel, G.E. Scuseria, M.A. Robb, J.R. Cheeseman, G. Scalmani, V. Barone, B.Mennucci, G.A. Petersson, H. Nakatsuji, M. Caricato, X. Li, H.P. Hratchian, A.F. Izmaylov, J. Bloino, G. Zheng, J.L. Sonnenberg, M. Hada, M. Ehara, K. Toyota, R.Fukuda, J. Hasegawa, M. Ishida, T. Nakajima, Y. Honda, O. Kitao, H. Nakai, T. Vreven, Jr., J.A. Montgomery J.E. Peralta, F. Ogliaro, M. Bearpark, J.J. Heyd, E, Brothers, K.N. Kudin, V.N. Staroverov, R. Kobayashi, J. Normand, K. Raghavachari, A. Rendell, J.C. Burant, S.S. Iyengar, J. Tomasi, M. Cossi, N. Rega, J.M. Millam, M. Klene, J.E. Knox, J.B. Cross, V. Bakken, C. Adamo, J. Jaramillo, R. Gomperts, R.E. Stratmann, O. Yazyev, A.J. Austin, R. Cammi, C. Pomelli, J.W. Ochterski, R.L. Martin, K. Morokuma, V.G. Zakrzewski, G.A. Voth, P. Salvador, J.J.Dannenberg, S. Dapprich, A.D. Daniels, O. Farkas, J.B. Foresman, J.V. Ortiz, J. Cioslowski, D.J.Fox, Gaussian 09, Revision A.02, Gaussian Inc., Pittsburgh, PA, 2009.

(4) G. Sheldrick, SHELXT - Integrated space-group and crystal-structure determination. *Acta Crystallographica Section A* 2015, **71**, 3-8.

(5) G. Sheldrick, Crystal structure refinement with SHELXL. *Acta Crystallographica Section C* 2015, **71**, 3-8.

(6) P. van der Sluis, A. L. Spek, BYPASS: an effective method for the refinement of crystal structures containing disordered solvent regions. *Acta Crystallographica Section A* 1999, **46**, 194-201.

(7) A. Spek, Single-crystal structure validation with the program PLATON. *Journal of Applied Crystallography* 2003, **36**, 7-13.
